# Supplementary material for: Trends in harmful drug exposure during pregnancy in France between 2013 and 2019: A nationwide cohort study
Source: PLoS One. 2024 Jan 10;19(1):e0295897. doi: 10.1371/journal.pone.0295897 (PMC10781191; doi:10.1371/journal.pone.0295897)
Supplement: S1 Table — (PDF) [file pone.0295897.s001.pdf]

S1 Table: Discharge codes or reimbursed drugs related to pregnancy outcomes and maternal conditions.

**Delivery**	
Diagnoses (CIM-10)	Principal diagnoses[a] O80 or O81 or O82 or O83 or O84<br>Or all diagnoses[b] Z37 or Z3900
Or procedure (CCAM[c])	JQGD001 to 005 or JQGD007 or JQGD008 or JQGD010 or JQGD012
**Abortion**	
Diagnoses (CIM-10)	Principal diagnoses O04 or O05 or O06 or O07
**Therapeutic abortion < 22 weeks' gestation**	
Codes for abortion	
Without codes for delivery	
Without diagnoses (CIM-10)	All diagnoses Z640
With procedure (CCAM)	JNJD001 or JNJD002 or JNJP001
**Therapeutic abortion ≥ 22 weeks' gestation**	
Codes for delivery	
With diagnoses (CIM-10)	Principal diagnoses (CIM-10) O04<br>Or all diagnoses: Z37.11 or Z37.31 or Z37.41 or Z37.61 or Z37.71
Or procedure (CCAM)	JNJD001 or JNJD002 or JNJP001
**Stillbirth**	
Codes for delivery	
With procedure (CCAM)	Z37.10 or Z37.30 or Z37.40 or Z37.60 or Z37.70
Without	Principal diagnoses (CIM-10) O04<br>Or procedure (CCAM) JNJD001 or JNJD002 or JNJP001
**Live birth**	
Codes for delivery	
Without codes for stillbirths	
Without codes for therapeutic abortion after 22 weeks' gestation	
**Spontaneous abortion**	
Diagnoses (CIM-10)	Principal diagnoses O03
Without codes for delivery	
**Ectopic pregnancy**	
Diagnoses (CIM-10)	Principal diagnoses O00
Or procedure (CCAM)	JJFA001 or JJFC001 or JJPA001 or JJPC001 or JJJA002 or JJJC002 or JJLJ001 or JQGA001
**Molar pregnancy or other abnormal products of conception**	
Diagnoses (CIM-10)	Principal diagnoses O01 or O02

**Psychiatric troubles (psychotic and humor disorders without addiction)**	
Psychiatric troubles (CIM-10)	F2, F30, F31, F32, F33, F34, F38, F39, F40, F41, F42, F43, F44, F45, F48
Drugs (ATC)	N05, N06

Pre-gestational diabetes	
Diabetes (CIM-10)	E10, E11, E12, E13, E14
Complication (CIM-10)	G59.0*, G63.2*, G73.0*, G99.0*, H28.0*, H36.0*, I79.2*, L97, M14.2*, M14.6*, N08.3*
Drugs (ATC)	A10AB01, A10AB03, A10AB04, A10AB05, A10AB06, A10AC01, A10AC03, A10AC04, A10AD01, A10AD03, A10AD04, A10AD05, A10AE01, A10AE02, A10AE03, A10AE04, A10AE05, A10AE30, A10AE54, A10AE56, A10BA02, A10BB01, A10BB03, A10BB04, A10BB06, A10BB07, A10BB09, A10BB12, A10BD02, A10BD03, A10BD05, A10BD07, A10BD08, A10BD10, A10BD15, A10BD16, A10BF01, A10BF02, A10BG02, A10BG03, A10BH01, A10BH02, A10BH03, A10BX02, A10BX04, A10BX07, A10BX09, A10BX10, A10BX11, A10BX12.

Hypertension	
Drugs (ATC)	C09C, C09D, C09A, C09B, C10BX04 ; C09X, C09DX02, C10BX04, C07B, C07CA03, C07DA06, C07FB02, C07FB03, C10BX03, C02A, C02B, C02C, C02D, C02K, C02L, C02N, C03A, C03B, C03C, C03D, C03E, C03X, C08C, C08D, C08E, C08G

Notes

Therapeutic abortion corresponds to termination of pregnancy for woman's request for fetal or maternal medical reasons. Stillbirth corresponds to death of a fetus with a gestational age ≥ 22 weeks'gestation or with a birth weight ≥ 500 g. Spontaneous abortion corresponds to fetal loss or death of a fetus with a gestational age < 22 weeks'gestation and a birth weight < 500g.

[a] Principal diagnoses correspond to patient's main illness during the hospital stay
[b] All diagnoses correspond to the principal diagnosis and the associated diagnosis
[c] CCAM (*Classifcation Commune des Actes Médicaux*) corresponds to the codes for medical procedures
