# Supplementary material for: Trends in harmful drug exposure during pregnancy in France between 2013 and 2019: A nationwide cohort study
Source: PLoS One. 2024 Jan 10;19(1):e0295897. doi: 10.1371/journal.pone.0295897 (PMC10781191; doi:10.1371/journal.pone.0295897)
Supplement: S2 Table — (PDF) [file pone.0295897.s002.pdf]

S2 Table: List of harmful drugs.

**Teratogenic drugs**	
**Antineoplasic and Immunomodulatory drugs**	fingolimod (L04AA27), leflunomide (L04AA13), lenalidomide (L04AX04), methotrexate (L04AX03, L01BA01), mycophenolic acid (L04AA06), thalidomide (L04AX02), teriflunomide (L04AA31)
**Retinoids for systemic use**	acitretin (for psoriasis treatment) (D05BB02), alitretinoin agents for dermatitis (D11AH04), etretinate (D05BB01), isotretinoin for systemic use (D10BA01), tretinoin (L01XX14)
**Retinoids for topical use**	adapalene (D10AD03, D10AD53), alitretinoin antineoplastic agent (L01XF02), isotretinoin (D10AD04), tifarotene (D10AD06), tretinoin (D10AD01, D10AD51)
**Antiepileptic drugs**	carbamazepine (N03AF01), fosphenytoin (N03AB05), oxcarbazepine (N03AF02), phenytoin (N03AB52), topiramate (N03AX11), valproic acid (N03AG01 without 3400934876233, 3400935444271, 3400934876691)
**Drugs for affective disorders**	valpromide (N03AG02), lithium (N05AN01), divalproate (3400934876233, 3400935444271, 3400934876691)
**Antithyroid preparations**	carbimazole (H03BB01), thiamazole (H03BB02)
**Vitamin K antagonists**	acenocoumarol (B01AA07), fluindione (3400933484132, 3400931183280, 3400931183112), warfarin (B01AA03)
**HMG CoA reductase inhibitors**	atorvastatin (C10AA05, C10BA06, C10BX03, C10BX06, C10BX11, C10BX15), fluvastatin (C10AA04), lovastatin (C10AA02), pitavastatin (C10AA08), pravastatin (C10AA03, C10BX02), rosuvastatin (C10AA07, C10BA06), simvastatin (C10AA01, C10BA02, C10BA04)
**Other drugs acting as teratogens**	acetazolamide, antiglaucoma preparation (S01EC01)

**Fetotoxic drugs**	
**Non-steroids anti-inflammatory drugs for systemic use**	
**Acetic acid derivatives and related substances**	aceclofenac (M01AB16), diclofenac (M01AB05, M01AB55), etodolac (M01AB08), indometacin (M01AB01), oxametacin (M01AB13), sulindac (M01AB02)
**Oxicams**	lornoxicam (M01AC05), meloxicam (M01AC06), piroxicam (M01AC01), tenoxicam (M01AC02)
**Propionic acid derivatives**	alminoprofen (M01AE16), dexketoprofen (M01AE17, N02AJ14), fenoprofen (M01AE04), flurbiprofen (M01AE09), ibuprofen (M01AE01, N02AJ08), ketoprofen (M01AE03), naproxen (M01AE02), tiaprofenic acid (M01AE11),
**Fenamate**	mefenamic acid (M01AG01)
**Coxibs**	celecoxib (M01AH01), etoricoxib (M01AH05)
**Other antiinflammatory and antirheumatic agents, non-steroids**	chondroitine sulfate (M01AX25), diacerein (M01AX21), feprazone (M01A26), glucosamine (M01AX05), morniflumate (M01AX22), nabumetone (M01AX01), niflumic acid (M01AX02)
**Acetylsalicylic acid (≥ 250mg)**	acetylsalicylic acid (3400934850097, 3400934300141, 3400933531539, 3400933588540, 3400933515102, 3400931898191, 3400932703616, 3400932610204, 3400931226895, 3400935146793, 3400935146854, 3400934735905, 3400935706720, 3400934736155, 3400930078181, 3400932062454, 3400932062515, 3400930076811, 3400931183402, 3400932213801, N02BA51, N02BA71)
**Non-steroids anti-inflammatory drugs for topical use**	
**Non-steroids anti-inflammatory drugs for topical use**	diclofenac (M02AA15), ibuprofen (M02AA13), ketoprofen (M02AA10), niflumic acid (M02AA17), piroxicam (M02AA07), various (M02AX10)
**Agents acting on the renin-angiotensin system**	
**Angiotensin Converting Enzyme (ACE) inhibitors**	captopril (C09AA01, C09BA01), benazetril (C09AA07, C09BA07), enalapril (C09AA02, C09BA02, C09BB02), fosinopril (C09AA09, C09BA09), lisinopril

	(C09AA03, C09BA03), perindopril (C09AA04, C09BA04, C09BB04, C09BX01, C09BX02), quinapril (C09AA06, C09BA06), ramipril (C09AA05,C09BA05), trandolapril (C09AA10, C09AB10), zofenopril (C09AA15, C09BA15)
**Angiotensin II receptor blockers**	candesartan (C09CA06, C09DA06), eprosartan (C09CA02), irbesartan (C09CA04, C09DA04), losartan (C09CA01, C09DA01), olmesartan (C09CA08, C09DA08, C09DB02), telmisartan (C09CA07, C09DA07, C09DB04), valsartan (C09CA03, C09DA03, C09DB01, C09DX01, C09DX04)
**Renin inhibitors**	aliskiren (C09XA02, C09XA52)

Contraceptives

**Hormonal contraceptives for systemic use**	Progestogens and estrogens, fixed combinations (G03AA01 to 17)<br>Progestogens and estrogens, sequential preparation (G03AB01 to 09)<br>Progestogens: desogestrel (G03AC09), etonogestrel (G03AC08), levonorgestrel (G03AC03), medroxyprogesterone (G03AC06)
**Emergency contraceptives**	levonorgestrel, emergency (G03D01), ulipristal (3400939450377, 3400930124086)
**Contraceptives for topical use**	plastic intrauterine device with progestogen (G02BA03, 3400930094754),<br>vaginal ring with progestogen and estrogen (G02BB01)

Sex hormones

**Androgens**	androstanolone (G03BB02), mesterolone (G03BB01), testosterone (G03BA03),
**Estrogens (for systemic use)**	estradiol (G03CA03), ethinylestradiol (G03CA01), tibolone (G03CX01)
**Estrogens (for topical use)**	estriol (G03CA04), promestriene (G03CA09)
**Progestogens**	chlormadinone (G03DB06), dienogest (G03DB08), lynestrenol (G03DC03), medrogestone (G03DB03), medroxyprogesterone (G03DA02), nomegestrol (G03DB04), norethisterone (G03DC02), promegestone (G03DB07)
**Anti-androgen**	cyproterone (G03HA01), cyproterone and estrogenes (G03HB01)
**Hormone replacement therapy**	Estrogen and progesteron with fixe combination (G03FA01 to 17)<br>Estrogen and progesteron with sequential preparation (G03FB01 to 12)
**Gonadotropins and other ovulation stimulants**	alfa follitropin (G03GA05), alfa lutropin (G03GA07), beta follitropin (G03GA06), chorionic gonadotrophin (G03GA01), human menopausal gonadotrophin (G03GA02), urofollitropin (G03GA04), clomifene (G03GB02)
**Antigonadotropins and similar agents**	danazol (G03XA01)
**Sex homones for systemic disease**	bazedoxifene (G03XC02), raloxifene (G03XC01), ulipristal (3400922257891, 3400927875304)
