# Supplementary material for: Trends in harmful drug exposure during pregnancy in France between 2013 and 2019: A nationwide cohort study
Source: PLoS One. 2024 Jan 10;19(1):e0295897. doi: 10.1371/journal.pone.0295897 (PMC10781191; doi:10.1371/journal.pone.0295897)
Supplement: S3 Table — Preconceptional period, T1, T2 and T3. (PDF) [file pone.0295897.s003.pdf]

S3 Table: : Maternal characteristics and pregnancy outcomes among pregnancies exposed to a teratogenic drug. Preconceptional period, T1, T2 and T3.

	During any time during pregnancy n=5,253,284	During preconceptional period or T1 n=5,253,284	During preconceptional period n=5,253,284	During T1 n=5,253,284	During T2 n=5,210,429	During T3 n=5,149,745
**Exposed pregnancies to a teratogenic drug, n (%)**	52,402 (1.0%)	48,326 (0.9%)	40,495 (0.8%)	17,773 (0.3%)	6,505 (0.1%)	5,294 (0.1%)
**Exposed pregnant women**	50,512	46,628	39,117	17,153	6,151	4,963
**Maternal age (years)**						
**Mean (+/- SD)**	30.8 +/- 5.5	30.9 +/- 5.5	30.9 +/- 5.5	31.4 +/- 5.8	31.6 +/- 5.9	31.6 +/- 5.5
**< 20**	808 (1.5%)	688 (1.4%)	515 (1.3%)	279 (1.6%)	136 (2.1%)	61 (1.2%)
**20-29**	21 083 (40.2%)	19,490 (40.3%)	16,161 (39.9%)	6,490 (36.5%)	2,217 (34.1%)	1,844 (34.8%)
**30-39**	27 097 (51.7%)	25,005 (51.7%)	21,151 (52.2%)	9,494 (53.4%)	3,566 (54.8%)	2,989 (56.5%)
**≥ 40**	3 414 (6.5%)	3,143 (6.5%)	2,668 (6.6%)	1,510 (8.5%)	586 (9.0%)	400 (7.6%)
**Maternal comorbidities**						
**Psychiatric troubles**	7 331 (14.0%)	7,002 (14.5%)	6,293 (15.5%)	3,191 (18.0%)	1,142 (17.6%)	1,049 (19.8%)
**Pre-gestational diabetes**	1 837 (3.5%)	1,764 (3.7%)	1,577 (3.9%)	831 (4.7%)	239 (3.7%)	111 (2.1%)
**Hypertension**	4 036 (7.7%)	3,886 (8.0%)	3,535 (8.7%)	1,776 (10.0%)	529 (8.1%)	341 (6.4%)
**Number of hospitalisations in the year prior to pregnancy**						
**Mean (+/- SD)**	0.6 +/- 1.9	0.6 +/- 1.9	0.6 +/- 1.8	0.6 +/- 2.3	0.6 +/- 3.2	0.5 +/- 1.4
**none**	36 219 (69.1%)	33,233 (68.8%)	27,579 (68.1%)	12,020 (67.6%)	4,453 (68.5%)	3,684 (69.6%)
**1**	10 445 (19.9%)	9,690 (20.1%)	8,224 (20.3%)	3,616 (20.4%)	1,329 (20.4%)	1,050 (19.8%)
**2 or more**	5 738 (11.0%)	5,403 (11.2%)	4,692 (11.6%)	2,137 (12.0%)	723 (11.1%)	560 (10.6%)
**Low-income status***	3 125 (6.0%)	2,812 (5.8%)	2,276 (5.6%)	1,108 (6.2%)	428 (6.6%)	290 (5.5%)
**Pregnancy outcome**						
**Live births**	50 508 (96.4%)	46,486 (96.2%)	38,910 (96.1%)	16,945 (95.3%)	6,334 (97.4%)	5,261 (99.4%)
**Medical termination <22GW**	1 274 (2.4%)	1,262 (2.6%)	1,086 (2.7%)	568 (3.2%)	46 (0.7%)	(0.0%)
**Medical termination≥22GW**	242 (0.5%)	231 (0.5%)	198 (0.5%)	114 (0.6%)	52 (0.8%)	12 (0.2%)
**Still births**	378 (0.7%)	347 (0.7%)	301 (0.7%)	146 (0.8%)	73 (1.1%)	21 (0.4%)
**Gestational age at birth (for live births only)**						
**Mean (+/- SD)**	38.8 +/- 2.1	38.8 +/- 2.1	38.8 +/- 2.1	38.7 +/- 2.2	39 +/- 1.7	39 +/- 1.7
**Premature birth < 37GW**	4 421 (8.4%)	4,133 (8.6%)	3,528 (8.7%)	1,672 (9.4%)	666 (10.2%)	359 (6.8%)
**<28GW**	237 (0.5%)	224 (0.5%)	195 (0.5%)	101 (0.6%)	41 (0.6%)	(0.0%)
**[28-31] GW**	487 (0.9%)	466 (1.0%)	409 (1.0%)	190 (1.1%)	72 (1.1%)	14 (0.3%)
**[32-36] GW**	3 697 (7.1%)	3,443 (7.1%)	2,924 (7.2%)	1,381 (7.8%)	553 (8.5%)	345 (6.5%)

*Low-income status was defined as affiliation to CMUc

Abbreviations: CMU (*couverture maladie universelle*), GW (gestational week), SD (standard deviation)

Data are shown as mean (+/- SD) or n (%)
