# Supplementary material for: Trends in harmful drug exposure during pregnancy in France between 2013 and 2019: A nationwide cohort study
Source: PLoS One. 2024 Jan 10;19(1):e0295897. doi: 10.1371/journal.pone.0295897 (PMC10781191; doi:10.1371/journal.pone.0295897)
Supplement: S4 Table — Number of pregnancies (rate per 10,000 pregnancies). (PDF) [file pone.0295897.s004.pdf]

S4 Table: Teratogenic drug exposure according to pregnancy period.

Number of pregnancies exposed during preconceptional period or T1(rate per 10,000 pregnancies)

	PC or T1	PC	T1	T2	T3	PC,T1,T2 or T3
**All identified pregnancies identified during the period**	**5,253,284**	**5,253,284**	**5,253,284**	**5,210,429**	**5,149,745**	**5,253,284**
**Pregnancies exposed to at least one teratogenic drug**						
**All teratogenic drugs**	**48,326 (92.0)**	**40,495 (77.1)**	**17,773 (33.8)**	**6,505 (12.5)**	**5,294 (10.3)**	**52,402 (99.8)**
**Antineoplasic and Immunomodulating**	**1,795 (3.4)**	**1,661 (3.2)**	**444 (0.8)**	**94 (0.2)**	**128 (0.2)**	**1,936 (3.7)**
mycophenolic acid	211 (0.4)	200 (0.4)	40 (0.1)	22 (0.0)	16 (0.0)	234 (0.4)
leflunomide	69 (0.1)	60 (0.1)	35 (0.1)	9 (0.0)	4 (0.0)	76 (0.1)
teriflunomide	72 (0.1)	65 (0.1)	31 (0.1)	2 (0.0)	14 (0.0)	84 (0.2)
fingolimod	203 (0.4)	199 (0.4)	111 (0.2)	6 (0.0)	18 (0.0)	214 (0.4)
methotrexate	1,243 (2.4)	1,138 (2.2)	227 (0.4)	55 (0.1)	76 (0.1)	1,331 (2.5)
**Retinoids for systemic use**	**737 (1.4)**	**660 (1.3)**	**135 (0.3)**	**40 (0.1)**	**22 (0.0)**	**778 (1.5)**
isotretinoin for systemic use	665 (1.3)	611 (1.2)	106 (0.2)	23 (0.0)	15 (0.0)	692 (1.3)
acitretin (retinoid psoriasis treatment)	58 (0.1)	38 (0.1)	24 (0.0)	15 (0.0)	6 (0.0)	72 (0.1)
alitretinoin	14 (0.0)	11 (0.0)	5 (0.0)	2 (0.0)	1 (0.0)	14 (0.0)
**Retinoids for topical use**	**22,863 (43.5)**	**18,233 (34.7)**	**6,032 (11.5)**	**1,380 (2.6)**	**1,031 (2.0)**	**24,718 (47.1)**
tretinoin	8,016 (15.3)	6,338 (12.1)	2,123 (4.0)	566 (1.1)	526 (1.0)	8,901 (16.9)
adapalene	15,254 (29.0)	12,183 (23.2)	3,985 (7.6)	824 (1.6)	514 (1.0)	16,266 (31.0)
**Antiepileptic drugs**	**6,635 (12.6)**	**5,894 (11.2)**	**3,809 (7.3)**	**2,410 (4.6)**	**2,243 (4.4)**	**7,220 (13.7)**
valproic acid	1,921 (3.7)	1,626 (3.1)	1,233 (2.3)	810 (1.6)	695 (1.3)	2,233 (4.3)
carbamazepin	1,926 (3.7)	1,697 (3.2)	1,275 (2.4)	952 (1.8)	922 (1.8)	2,086 (4.0)
oxcarbazepin	533 (1.0)	465 (0.9)	430 (0.8)	382 (0.7)	380 (0.7)	598 (1.1)
phenytoin	18 (0.0)	16 (0.0)	13 (0.0)	19 (0.0)	17 (0.0)	30 (0.1)
topiramate	2,416 (4.6)	2,230 (4.2)	976 (1.9)	323 (0.6)	294 (0.6)	2,482 (4.7)
**Drugs for affective disorders**	**2,789 (5.3)**	**2,542 (4.8)**	**1,288 (2.5)**	**518 (1.0)**	**568 (1.1)**	**2,955 (5.6)**
valpromide	879 (1.7)	790 (1.5)	344 (0.7)	64 (0.1)	64 (0.1)	904 (1.7)
lithium	991 (1.9)	924 (1.8)	569 (1.1)	388 (0.7)	460 (0.9)	1,108 (2.1)
divalproate	973 (1.9)	872 (1.7)	387 (0.7)	66 (0.1)	45 (0.1)	1,008 (1.9)
**Antithyroid preparations**	**3,876 (7.4)**	**3,120 (5.9)**	**1,923 (3.7)**	**963 (1.8)**	**649 (1.3)**	**4,549 (8.7)**
thiamazole	1,387 (2.6)	1,179 (2.2)	628 (1.2)	219 (0.4)	145 (0.3)	1,523 (2.9)
carbimazole	2,575 (4.9)	1,991 (3.8)	1,313 (2.5)	750 (1.4)	509 (1.0)	3,170 (6.0)
**Vitamin K antagonists**	**2,490 (4.7)**	**2,319 (4.4)**	**1,257 (2.4)**	**258 (0.5)**	**142 (0.3)**	**2,591 (4.9)**
warfarin	856 (1.6)	803 (1.5)	447 (0.9)	92 (0.2)	68 (0.1)	900 (1.7)
acenocoumarol	139 (0.3)	117 (0.2)	75 (0.1)	41 (0.1)	17 (0.0)	156 (0.3)
fluindione	1,526 (2.9)	1,414 (2.7)	745 (1.4)	125 (0.2)	57 (0.1)	1,576 (3.0)
**HMG Coa reductase inhibitors**	**6,621 (12.6)**	**5,591 (10.6)**	**2,726 (5.2)**	**768 (1.5)**	**440 (0.9)**	**7,149 (13.6)**
simvastatin	1,141 (2.2)	968 (1.8)	456 (0.9)	128 (0.2)	70 (0.1)	1,247 (2.4)
simvastatin and ezetimibe	269 (0.5)	229 (0.4)	106 (0.2)	24 (0.0)	11 (0.0)	288 (0.5)
pravastatin	720 (1.4)	619 (1.2)	278 (0.5)	89 (0.2)	39 (0.1)	787 (1.5)
pravastatin and acetylsalicylic acid	13 (0.0)	12 (0.0)	8 (0.0)	1 (0.0)	(0.0)	13 (0.0)
fluvastatin	122 (0.2)	106 (0.2)	45 (0.1)	6 (0.0)	6 (0.0)	126 (0.2)
atorvastatin	2,796 (5.3)	2,312 (4.4)	1,214 (2.3)	330 (0.6)	192 (0.4)	3,016 (5.7)
atorvastatin and ezetimibe	93 (0.2)	82 (0.2)	34 (0.1)	11 (0.0)	8 (0.0)	105 (0.2)
atorvastatin and amlodipin	14 (0.0)	12 (0.0)	4 (0.0)	8 (0.0)	3 (0.0)	19 (0.0)
rosuvastatin	1,543 (2.9)	1,298 (2.5)	599 (1.1)	180 (0.3)	112 (0.2)	1,660 (3.2)
**Other drugs acting as teratogens**	**1,120 (2.1)**	**941 (1.8)**	**330 (0.6)**	**118 (0.2)**	**105 (0.2)**	**1,208 (2.3)**
acetazolamide	1,120 (2.1)	941 (1.8)	330 (0.6)	118 (0.2)	105 (0.2)	1,208 (2.3)
