# Supplementary material for: Trends in harmful drug exposure during pregnancy in France between 2013 and 2019: A nationwide cohort study
Source: PLoS One. 2024 Jan 10;19(1):e0295897. doi: 10.1371/journal.pone.0295897 (PMC10781191; doi:10.1371/journal.pone.0295897)
Supplement: S5 Table — Number of pregnancies (rate per 10,000 pregnancies). (PDF) [file pone.0295897.s005.pdf]

S5 Table: Foetotoxic drug exposure according to pregnancy period.

Number of pregnancies exposed during T2 or T3 (rate per 10,000 pregnancies)

	T2 or T3	T2	T3
**All pregnancies identified during the period**	**5,210,429**	**5,210,429**	**5,149,745**
**Pregnancies exposed to at least one foetotoxic drug**			
**All foetotoxic drugs**	**155,514 (298.5)**	**94,740 (181.8)**	**67,998 (132.0)**
**Non-steroids anti-inflammatory drugs for systemic use**	**66,662 (127.9)**	**43,747 (84.0)**	**25,050 (48.6)**
**Acetic acid derivatives and related substances**			
indometacin (systemic use)	386 (0.7)	358 (0.7)	30 (0.1)
sulindac	10 (0.0)	9 (0.0)	1 (0.0)
diclofenac (systemic use)	4,414 (8.5)	3,231 (6.2)	1,342 (2.6)
diclofenac (combinaison)	131 (0.3)	95 (0.2)	40 (0.1)
etodolac	94 (0.2)	74 (0.1)	22 (0.0)
aceclofenac (systemic use)	297 (0.6)	216 (0.4)	83 (0.2)
**Oxicams**			
piroxicam (systemic use)	1,010 (1.9)	770 (1.5)	261 (0.5)
tenoxicam	116 (0.2)	85 (0.2)	33 (0.1)
meloxicam	155 (0.3)	107 (0.2)	53 (0.1)
**Propionic acid derivatives**			
ibuprofen (systemic use)	34,377 (66.0)	21,383 (41.0)	13,690 (26.6)
naproxen (systemic use)	2,280 (4.4)	1,598 (3.1)	720 (1.4)
ketoprofen (systemic use)	10,743 (20.6)	6,523 (12.5)	4,392 (8.5)
fenoprofen	57 (0.1)	37 (0.1)	22 (0.0)
flurbiprofen (ystemique)	3,078 (5.9)	2,084 (4.0)	1,088 (2.1)
tiaprofrenic acid	3,704 (7.1)	2,672 (5.1)	1,072 (2.1)
alminoprofen	10 (0.0)	7 (0.0)	3 (0.0)
**Fenamates**			
mefenamic acid	318 (0.6)	210 (0.4)	115 (0.2)
**Coxibs**			
celecoxib	328 (0.6)	239 (0.5)	107 (0.2)
etoricoxib	149 (0.3)	95 (0.2)	62 (0.1)
**Other antiinflammatory and antirheumatic agents**			
nabumetone	242 (0.5)	181 (0.3)	62 (0.1)
niflumic acid (systemic use)	1,120 (2.1)	804 (1.5)	319 (0.6)
glucosamine	51 (0.1)	40 (0.1)	20 (0.0)
feprazone (combinations)	433 (0.8)	328 (0.6)	149 (0.3)
diacerein	9 (0.0)	4 (0.0)	5 (0.0)
morniflumate	1,230 (2.4)	836 (1.6)	397 (0.8)
chondroitin sulfate	84 (0.2)	60 (0.1)	37 (0.1)
**Acetylsalicylic Acid**			
acetylsalicylic acid	4,909 (9.4)	3,477 (6.7)	1,592 (3.1)
**Non-steroids anti-inflammatory drugs for topical use**	**63,330 (121.5)**	**43,436 (83.4)**	**22,484 (43.7)**
piroxicam (topical use)	756 (1.5)	519 (1.0)	248 (0.5)
ketoprofen (topical use)	2,221 (4.3)	1,552 (3.0)	724 (1.4)
ibuprofen (topical use)	9,219 (17.7)	6,428 (12.3)	2,982 (5.8)
diclofenac (topical use)	44,754 (85.9)	30,569 (58.7)	15,963 (31.0)
niflumic acid (topical use)	7,613 (14.6)	4,956 (9.5)	2,793 (5.4)
others	43 (0.1)	31 (0.1)	12 (0.0)
**Agents acting on the renin-angiotensin system**	**2,303 (4.4)**	**1,672 (3.2)**	**936 (1.8)**
**Angiotensin Converting Enzyme inhibitors**			
captopril	47 (0.1)	34 (0.1)	17 (0.0)
captopril and diuretics	17 (0.0)	12 (0.0)	6 (0.0)
enalapril	83 (0.2)	46 (0.1)	49 (0.1)
enalapril and diuretics	22 (0.0)	15 (0.0)	9 (0.0)
enalapril andlecarnidipin	41 (0.1)	34 (0.1)	16 (0.0)
lisinopril	25 (0.0)	17 (0.0)	11 (0.0)
lisinopriland and diuretics	7 (0.0)	5 (0.0)	4 (0.0)
perindopril	234 (0.4)	179 (0.3)	83 (0.2)
perindopril and diuretics	118 (0.2)	79 (0.2)	48 (0.1)
perindopril and amlodipine	178 (0.3)	148 (0.3)	63 (0.1)
perindopril and bisoprolol	1 (0.0)	1 (0.0)	(0.0)
ramipril	290 (0.6)	206 (0.4)	113 (0.2)
ramipril and diuretics	30 (0.1)	23 (0.0)	15 (0.0)
quinapril	3 (0.0)	2 (0.0)	1 (0.0)
quinapril and diuretics	6 (0.0)	3 (0.0)	5 (0.0)
benazepril	15 (0.0)	13 (0.0)	6 (0.0)
benazepril and diuretics	(0.0)	(0.0)	(0.0)
fosinopril	6 (0.0)	2 (0.0)	4 (0.0)
fosinopriland and diuretics	2 (0.0)	1 (0.0)	1 (0.0)
trandolapril	20 (0.0)	12 (0.0)	10 (0.0)
trandolapril and diuretics	21 (0.0)	15 (0.0)	11 (0.0)
zofenopril	6 (0.0)	3 (0.0)	3 (0.0)

zofenopriland and diuretics	1 (0.0)	(0.0)	1 (0.0)
**Angiotensin II receptor blockers**			
losartan	94 (0.2)	70 (0.1)	36 (0.1)
losartan and diuretics	66 (0.1)	41 (0.1)	31 (0.1)
eprosartan	1 (0.0)	1 (0.0)	(0.0)
valsartan	73 (0.1)	53 (0.1)	27 (0.1)
valsartan and diuretics	92 (0.2)	73 (0.1)	30 (0.1)
valsartan and amlodipine	136 (0.3)	100 (0.2)	51 (0.1)
irbesartan	167 (0.3)	128 (0.2)	57 (0.1)
irbesartan and diuretics	135 (0.3)	96 (0.2)	57 (0.1)
candesartan	128 (0.2)	79 (0.2)	63 (0.1)
candesartan and diuretics	58 (0.1)	40 (0.1)	29 (0.1)
telmisartan	55 (0.1)	39 (0.1)	23 (0.0)
telmisartan and diuretics	40 (0.1)	27 (0.1)	17 (0.0)
temisartan and amlodipine	14 (0.0)	13 (0.0)	6 (0.0)
olmesartan medoxomil	53 (0.1)	43 (0.1)	13 (0.0)
olmesartan meoxomilol and diuretics	25 (0.0)	18 (0.0)	11 (0.0)
olmesartan medoxomil and amlodipine	38 (0.1)	30 (0.1)	15 (0.0)
**Renin inhibitors**			
aliskiren	9 (0.0)	6 (0.0)	5 (0.0)
aliskiren et hydrochlorothiazide	4 (0.0)	2 (0.0)	3 (0.0)
**Contraceptives**	**22,315 (42.8)**	**5,138 (9.9)**	**17,424 (33.8)**
**Hormonal contraceptives for systemic use**			
norandhisterone and ethinylestradiol	25 (0.0)	16 (0.0)	10 (0.0)
norgestrel and ethinylestradiol	37 (0.1)	32 (0.1)	5 (0.0)
levonorgestrel and ethinylestradiol	4,656 (8.9)	3,405 (6.5)	1,420 (2.8)
desogestrel and ethinylestradiol	1 (0.0)	1 (0.0)	(0.0)
gestoden and ethinylestradiol	1 (0.0)	1 (0.0)	(0.0)
etonogestrel	11,533 (22.1)	553 (1.1)	10,985 (21.3)
desogestrel and ethinylestradiol	4,785 (9.2)	757 (1.5)	4,054 (7.9)
**Emergency contraceptives**			
levonorgestrel (emergency)	696 (1.3)	291 (0.6)	408 (0.8)
ulipristal (emergency)	61 (0.1)	32 (0.1)	29 (0.1)
**Contraceptives for topical use**			
DIU with progestogen	884 (1.7)	169 (0.3)	716 (1.4)
**Sex hormones**	**12,840 (24.6)**	**8,043 (15.4)**	**5,387 (10.5)**
**Androgens**			
testosterone	12 (0.0)	10 (0.0)	5 (0.0)
androstanolone	6 (0.0)	5 (0.0)	2 (0.0)
**Estrogens (for systemic use)**			
estradiol	872 (1.7)	746 (1.4)	145 (0.3)
**Estrogens (for topical use)**			
estriol	644 (1.2)	383 (0.7)	285 (0.6)
promestrien	1,907 (3.7)	1,180 (2.3)	768 (1.5)
**Progestogens**			
medrogestone	4 (0.0)	4 (0.0)	(0.0)
nomegestrol	207 (0.4)	160 (0.3)	61 (0.1)
chlormadinone	134 (0.3)	108 (0.2)	30 (0.1)
promegestone	40 (0.1)	34 (0.1)	6 (0.0)
**Anti-androgen**			
cyproterone	59 (0.1)	38 (0.1)	24 (0.0)
cyproterone and estrogens	1 (0.0)	(0.0)	1 (0.0)
**Hormone replacement therapy**			
norandhisterone and esotrogens	4 (0.0)	4 (0.0)	(0.0)
progesterone and estrogens	8,522 (16.4)	5,030 (9.7)	3,918 (7.6)
medroxyprogesterone and estrogens	1 (0.0)	(0.0)	1 (0.0)
dydrogesterone and estrogens	7 (0.0)	4 (0.0)	4 (0.0)
norandhisterone and estrogens (sequential preparation)	1 (0.0)	1 (0.0)	(0.0)
medroxyprogesterone and estrogens (sequential preparation)	1 (0.0)	1 (0.0)	(0.0)
dydrogesterone and estrogens (sequential preparation)	22 (0.0)	16 (0.0)	8 (0.0)
**Gonadotropins and other ovulation stimulants**			
chorionic gonadotrophin	15 (0.0)	13 (0.0)	3 (0.0)
human menopausal gonadotrophin	65 (0.1)	51 (0.1)	23 (0.0)
urofollitropin	7 (0.0)	5 (0.0)	2 (0.0)
follitropin alfa	86 (0.2)	66 (0.1)	22 (0.0)
follitropin beta	27 (0.1)	18 (0.0)	11 (0.0)
lutropin alfa	1 (0.0)	1 (0.0)	(0.0)
choriogonadotropin alfa	100 (0.2)	72 (0.1)	31 (0.1)
clomifene	325 (0.6)	258 (0.5)	87 (0.2)
**Antigonadotropins and similar agents**			
danazol	7 (0.0)	5 (0.0)	2 (0.0)
**Sex hormones for systemic disease**			
raloxifen	5 (0.0)	5 (0.0)	(0.0)
ulipristal (ESMYA)	23 (0.0)	14 (0.0)	12 (0.0)
