# Supplementary material for: Trends in harmful drug exposure during pregnancy in France between 2013 and 2019: A nationwide cohort study
Source: PLoS One. 2024 Jan 10;19(1):e0295897. doi: 10.1371/journal.pone.0295897 (PMC10781191; doi:10.1371/journal.pone.0295897)
Supplement: S6 Table — Number of pregnancies (rate per 10,000 pregnancies). (PDF) [file pone.0295897.s006.pdf]

S6 Table: Trends in teratogenic drug exposure during pregnancy between 2013 and 2019.

Number of pregnancies exposed during preconceptional period or T1 (rate per 10,000 pregnancies)

	2013	2014	2015	2016	2017	2018	2019
**All pregnancies identified during the period**	**790,664**	**774,640**	**764,881**	**746,832**	**736,475**	**726,083**	**713,709**
**Pregnancies exposed to at least one teratogenic drug**							
**All teratogenic drugs**	**8,898 (112.5)**	**8,488 (109.6)**	**7,309 (95.6)**	**6,556 (87.8)**	**6,069 (82.4)**	**5,772 (79.5)**	**5,234 (73.3)**
**Antineoplasic and Immunomodulating**	**202 (2.6)**	**256 (3.3)**	**282 (3.7)**	**258 (3.5)**	**241 (3.3)**	**274 (3.8)**	**282 (4.0)**
mycophenolic acid	25 (0.3)	42 (0.5)	33 (0.4)	34 (0.5)	24 (0.3)	25 (0.3)	28 (0.4)
leflunomide	8 (0.1)	8 (0.1)	11 (0.1)	13 (0.2)	9 (0.1)	12 (0.2)	8 (0.1)
teriflunomide	(0.0)	1 (0.0)	12 (0.2)	15 (0.2)	14 (0.2)	15 (0.2)	15 (0.2)
fingolimod	12 (0.2)	30 (0.4)	28 (0.4)	27 (0.4)	34 (0.5)	29 (0.4)	43 (0.6)
methotrexate	157 (2.0)	175 (2.3)	199 (2.6)	169 (2.3)	161 (2.2)	194 (2.7)	188 (2.6)
**Retinoids for systemic use**	**107 (1.4)**	**119 (1.5)**	**115 (1.5)**	**111 (1.5)**	**101 (1.4)**	**103 (1.4)**	**81 (1.1)**
isotretinoin for systemic use	90 (1.1)	108 (1.4)	98 (1.3)	102 (1.4)	93 (1.3)	99 (1.4)	75 (1.1)
acitretin (retinoid psoriasis treatment)	14 (0.2)	10 (0.1)	13 (0.2)	8 (0.1)	6 (0.1)	2 (0.0)	5 (0.1)
alitretinoin	3 (0.0)	1 (0.0)	4 (0.1)	1 (0.0)	2 (0.0)	2 (0.0)	1 (0.0)
**Retinoids for topical use**	**4,200 (53.1)**	**4,098 (52.9)**	**3,373 (44.1)**	**3,079 (41.2)**	**2,927 (39.7)**	**2,754 (37.9)**	**2,432 (34.1)**
tretinoin	1,543 (19.5)	1,539 (19.9)	1,182 (15.5)	1,039 (13.9)	975 (13.2)	922 (12.7)	816 (11.4)
adapalene	2,765 (35.0)	2,639 (34.1)	2,240 (29.3)	2,088 (28.0)	2,009 (27.3)	1,868 (25.7)	1,645 (23.0)
**Antiepileptic drugs**	**1,300 (16.4)**	**1,253 (16.2)**	**1,018 (13.3)**	**904 (12.1)**	**727 (9.9)**	**761 (10.5)**	**672 (9.4)**
valproic acid	532 (6.7)	464 (6.0)	349 (4.6)	228 (3.1)	128 (1.7)	137 (1.9)	83 (1.2)
carbamazepin	337 (4.3)	314 (4.1)	287 (3.8)	286 (3.8)	231 (3.1)	236 (3.3)	235 (3.3)
oxcarbazepin	75 (0.9)	91 (1.2)	81 (1.1)	82 (1.1)	67 (0.9)	71 (1.0)	66 (0.9)
phenytoin	7 (0.1)	4 (0.1)	(0.0)	(0.0)	3 (0.0)	3 (0.0)	1 (0.0)
topiramate	406 (5.1)	406 (5.2)	330 (4.3)	332 (4.4)	310 (4.2)	327 (4.5)	305 (4.3)
**Drugs for affective disorders**	**668 (8.4)**	**642 (8.3)**	**499 (6.5)**	**314 (4.2)**	**238 (3.2)**	**229 (3.2)**	**199 (2.8)**
valpromide	238 (3.0)	247 (3.2)	170 (2.2)	91 (1.2)	70 (1.0)	44 (0.6)	19 (0.3)
lithium	146 (1.8)	139 (1.8)	138 (1.8)	140 (1.9)	126 (1.7)	149 (2.1)	153 (2.1)
divalproate	297 (3.8)	270 (3.5)	201 (2.6)	90 (1.2)	47 (0.6)	37 (0.5)	31 (0.4)
**Antithyroid preparations**	**581 (7.3)**	**501 (6.5)**	**544 (7.1)**	**578 (7.7)**	**544 (7.4)**	**601 (8.3)**	**527 (7.4)**
thiamazole	149 (1.9)	278 (3.6)	216 (2.8)	188 (2.5)	186 (2.5)	200 (2.8)	170 (2.4)
carbimazole	457 (5.8)	251 (3.2)	337 (4.4)	397 (5.3)	365 (5.0)	407 (5.6)	361 (5.1)
**Vitamin K antagonists**	**529 (6.7)**	**451 (5.8)**	**406 (5.3)**	**307 (4.1)**	**333 (4.5)**	**241 (3.3)**	**223 (3.1)**
warfarin	132 (1.7)	120 (1.5)	127 (1.7)	117 (1.6)	139 (1.9)	104 (1.4)	117 (1.6)
acenocoumarol	23 (0.3)	31 (0.4)	24 (0.3)	17 (0.2)	20 (0.3)	13 (0.2)	11 (0.2)
fluindione	380 (4.8)	307 (4.0)	260 (3.4)	176 (2.4)	179 (2.4)	128 (1.8)	96 (1.3)
**HMG Coa reductase inhibitors**	**1,286 (16.3)**	**1,164 (15.0)**	**1,018 (13.3)**	**907 (12.1)**	**856 (11.6)**	**690 (9.5)**	**700 (9.8)**
simvastatin	211 (2.7)	199 (2.6)	184 (2.4)	172 (2.3)	148 (2.0)	106 (1.5)	121 (1.7)
simvastatin and ezetimibe	59 (0.7)	54 (0.7)	47 (0.6)	39 (0.5)	27 (0.4)	25 (0.3)	18 (0.3)
pravastatin	143 (1.8)	123 (1.6)	119 (1.6)	92 (1.2)	94 (1.3)	73 (1.0)	76 (1.1)
pravastatin and acetylsalicylic acid	11 (0.1)	2 (0.0)	(0.0)	(0.0)	(0.0)	(0.0)	(0.0)
fluvastatin	28 (0.4)	19 (0.2)	26 (0.3)	15 (0.2)	14 (0.2)	12 (0.2)	8 (0.1)
atorvastatin	409 (5.2)	426 (5.5)	419 (5.5)	414 (5.5)	423 (5.7)	348 (4.8)	357 (5.0)
atorvastatin and ezetimibe	(0.0)	(0.0)	(0.0)	14 (0.2)	29 (0.4)	25 (0.3)	25 (0.4)
atorvastatin and amlodipin	1 (0.0)	2 (0.0)	3 (0.0)	2 (0.0)	2 (0.0)	2 (0.0)	2 (0.0)
rosuvastatin	439 (5.6)	351 (4.5)	234 (3.1)	170 (2.3)	135 (1.8)	111 (1.5)	103 (1.4)
**Other drugs acting as teratogens**	**140 (1.8)**	**155 (2.0)**	**148 (1.9)**	**175 (2.3)**	**162 (2.2)**	**169 (2.3)**	**171 (2.4)**
acetazolamide	140 (1.8)	155 (2.0)	148 (1.9)	175 (2.3)	162 (2.2)	169 (2.3)	171 (2.4)
