# Supplementary material for: Trends in harmful drug exposure during pregnancy in France between 2013 and 2019: A nationwide cohort study
Source: PLoS One. 2024 Jan 10;19(1):e0295897. doi: 10.1371/journal.pone.0295897 (PMC10781191; doi:10.1371/journal.pone.0295897)
Supplement: S7 Table — Number of pregnancies (rate per 10,000 pregnancies). (PDF) [file pone.0295897.s007.pdf]

S7 Table: Trends in foetotoxic drug exposure during pregnancy between 2013 and 2019.

Number of pregnancies exposed during T2 or T3 (rate per 10,000 pregnancies)

	2013	2014	2015	2016	2017	2018	2019
**All pregnancies identified during the period**	**784,057**	**768,143**	**758,598**	**740,743**	**730,834**	**720,026**	**708,028**
**Pregnancies exposed to at least one foetotoxic drug**							
**All foetotoxic drugs**	**27,188 (346.8)**	**26,866 (349.8)**	**24,903 (328.3)**	**22,207 (299.8)**	**20,034 (274.1)**	**18,388 (255.4)**	**15,928 (225.0)**
**Non-steroids anti-inflammatory drugs for systemic use**	**11,266 (143.7)**	**11,323 (147.4)**	**10,672 (140.7)**	**9,507 (128.3)**	**8,794 (120.3)**	**8,290 (115.1)**	**6,810 (96.2)**
**Acetic acid derivatives and related substances**							
indometacin (systemic use)	66 (0.8)	62 (0.8)	71 (0.9)	57 (0.8)	44 (0.6)	44 (0.6)	42 (0.6)
sulindac	(0.0)	3 (0.0)	2 (0.0)	2 (0.0)	(0.0)	2 (0.0)	1 (0.0)
diclofenac (systemic use)	758 (9.7)	754 (9.8)	690 (9.1)	667 (9.0)	591 (8.1)	519 (7.2)	435 (6.1)
diclofenac (combinaison)	36 (0.5)	26 (0.3)	25 (0.3)	24 (0.3)	14 (0.2)	6 (0.1)	(0.0)
etodolac	29 (0.4)	16 (0.2)	13 (0.2)	16 (0.2)	8 (0.1)	7 (0.1)	5 (0.1)
aceclofenac (systemic use)	66 (0.8)	48 (0.6)	39 (0.5)	43 (0.6)	37 (0.5)	34 (0.5)	30 (0.4)
**Oxicams**							
piroxicam (systemic use)	196 (2.5)	180 (2.3)	188 (2.5)	134 (1.8)	119 (1.6)	104 (1.4)	89 (1.3)
tenoxicam	19 (0.2)	17 (0.2)	13 (0.2)	24 (0.3)	23 (0.3)	9 (0.1)	11 (0.2)
meloxicam	26 (0.3)	26 (0.3)	17 (0.2)	25 (0.3)	23 (0.3)	22 (0.3)	16 (0.2)
**Propionic acid derivatives**							
ibuprofen (systemic use)	5,241 (66.8)	5,658 (73.7)	5,574 (73.5)	5,041 (68.1)	4,719 (64.6)	4,530 (62.9)	3,614 (51.0)
naproxen (systemic use)	389 (5.0)	395 (5.1)	363 (4.8)	332 (4.5)	287 (3.9)	273 (3.8)	241 (3.4)
ketoprofen (systemic use)	1,779 (22.7)	1,724 (22.4)	1,635 (21.6)	1,527 (20.6)	1,398 (19.1)	1,429 (19.8)	1,251 (17.7)
fenoprofen	16 (0.2)	8 (0.1)	10 (0.1)	7 (0.1)	7 (0.1)	8 (0.1)	1 (0.0)
flurbiprofen (ystemique)	514 (6.6)	535 (7.0)	496 (6.5)	400 (5.4)	418 (5.7)	363 (5.0)	352 (5.0)
tiaprofrenic acid	760 (9.7)	675 (8.8)	645 (8.5)	481 (6.5)	465 (6.4)	402 (5.6)	276 (3.9)
alminoprofen	2 (0.0)	1 (0.0)	1 (0.0)	3 (0.0)	(0.0)	2 (0.0)	1 (0.0)
**Fenamates**							
mefenamic acid	54 (0.7)	63 (0.8)	53 (0.7)	54 (0.7)	23 (0.3)	26 (0.4)	45 (0.6)
**Coxibs**							
celecoxib	62 (0.8)	52 (0.7)	40 (0.5)	42 (0.6)	46 (0.6)	42 (0.6)	44 (0.6)
etoricoxib	34 (0.4)	21 (0.3)	25 (0.3)	26 (0.4)	18 (0.2)	15 (0.2)	10 (0.1)
**Other antiinflammatory and antirheumatic agents**							
nabumetone	43 (0.5)	41 (0.5)	40 (0.5)	33 (0.4)	29 (0.4)	33 (0.5)	23 (0.3)
niflumic acid (systemic use)	233 (3.0)	241 (3.1)	200 (2.6)	135 (1.8)	136 (1.9)	95 (1.3)	80 (1.1)
glucosamine	30 (0.4)	21 (0.3)	(0.0)	(0.0)	(0.0)	(0.0)	(0.0)
feprazone (combinations)	248 (3.2)	185 (2.4)	(0.0)	(0.0)	(0.0)	(0.0)	(0.0)
diacerein	7 (0.1)	2 (0.0)	(0.0)	(0.0)	(0.0)	(0.0)	(0.0)
morniflumate	249 (3.2)	232 (3.0)	207 (2.7)	169 (2.3)	143 (2.0)	132 (1.8)	98 (1.4)
chondroitin sulfate	38 (0.5)	44 (0.6)	(0.0)	(0.0)	1 (0.0)	(0.0)	1 (0.0)
**Acetylsalicylic Acid**							
acetylsalicylic acid	938 (12.0)	862 (11.2)	803 (10.6)	711 (9.6)	629 (8.6)	535 (7.4)	431 (6.1)
**Non-steroids anti-inflammatory drugs for topical use**	**12,052 (153.7)**	**11,544 (150.3)**	**10,223 (134.8)**	**8,987 (121.3)**	**7,876 (107.8)**	**6,672 (92.7)**	**5,976 (84.4)**
piroxicam (topical use)	164 (2.1)	153 (2.0)	106 (1.4)	110 (1.5)	95 (1.3)	69 (1.0)	59 (0.8)
ketoprofen (topical use)	1,333 (17.0)	842 (11.0)	18 (0.2)	8 (0.1)	11 (0.2)	5 (0.1)	4 (0.1)
ibuprofen (topical use)	564 (7.2)	1,319 (17.2)	1,612 (21.2)	1,656 (22.4)	1,501 (20.5)	1,359 (18.9)	1,208 (17.1)
diclofenac (topical use)	8,680 (110.7)	7,893 (102.8)	7,374 (97.2)	6,275 (84.7)	5,556 (76.0)	4,706 (65.4)	4,270 (60.3)
niflumic acid (topical use)	1,598 (20.4)	1,587 (20.7)	1,271 (16.8)	1,097 (14.8)	853 (11.7)	671 (9.3)	536 (7.6)
others	0 (0.0)	5 (0.1)	19 (0.3)	8 (0.1)	9 (0.1)	2 (0.0)	0 (0.0)
**Agents acting on the renin-angiotensin system**	**346 (4.4)**	**371 (4.8)**	**372 (4.9)**	**325 (4.4)**	**314 (4.3)**	**296 (4.1)**	**279 (3.9)**
**Angiotensin Converting Enzyme inhibitors**							
captopril	7 (0.1)	10 (0.1)	4 (0.1)	9 (0.1)	3 (0.0)	9 (0.1)	5 (0.1)
captopril and diuretics	2 (0.0)	4 (0.1)	1 (0.0)	3 (0.0)	2 (0.0)	4 (0.1)	1 (0.0)
enalapril	12 (0.2)	13 (0.2)	9 (0.1)	11 (0.1)	14 (0.2)	13 (0.2)	11 (0.2)
enalapril and diuretics	2 (0.0)	2 (0.0)	6 (0.1)	5 (0.1)	2 (0.0)	2 (0.0)	3 (0.0)
enalapril andlecarnidipin	4 (0.1)	3 (0.0)	12 (0.2)	10 (0.1)	7 (0.1)	3 (0.0)	2 (0.0)
lisinopril	4 (0.1)	3 (0.0)	2 (0.0)	6 (0.1)	5 (0.1)	1 (0.0)	4 (0.1)
lisinopriland and diuretics	0 (0.0)	1 (0.0)	1 (0.0)	0 (0.0)	2 (0.0)	2 (0.0)	1 (0.0)
perindopril	40 (0.5)	33 (0.4)	37 (0.5)	36 (0.5)	29 (0.4)	27 (0.4)	32 (0.5)
perindopril and diuretics	21 (0.3)	19 (0.2)	23 (0.3)	19 (0.3)	14 (0.2)	9 (0.1)	13 (0.2)
perindopril and amlodipine	15 (0.2)	26 (0.3)	35 (0.5)	17 (0.2)	29 (0.4)	30 (0.4)	26 (0.4)
perindopril and bisoprolol	0 (0.0)	0 (0.0)	0 (0.0)	0 (0.0)	0 (0.0)	1 (0.0)	0 (0.0)
ramipril	47 (0.6)	38 (0.5)	43 (0.6)	38 (0.5)	46 (0.6)	33 (0.5)	45 (0.6)
ramipril and diuretics	1 (0.0)	4 (0.1)	4 (0.1)	4 (0.1)	5 (0.1)	6 (0.1)	6 (0.1)
quinapril	1 (0.0)	1 (0.0)	0 (0.0)	0 (0.0)	0 (0.0)	0 (0.0)	1 (0.0)
quinapril and diuretics	0 (0.0)	3 (0.0)	1 (0.0)	0 (0.0)	0 (0.0)	2 (0.0)	0 (0.0)
benazepril	3 (0.0)	4 (0.1)	2 (0.0)	1 (0.0)	3 (0.0)	0 (0.0)	2 (0.0)
benazepril and diuretics	0 (0.0)	0 (0.0)	0 (0.0)	0 (0.0)	0 (0.0)	0 (0.0)	0 (0.0)
fosinopril	0 (0.0)	1 (0.0)	2 (0.0)	(0.0)	1 (0.0)	1 (0.0)	1 (0.0)
fosinopriland and diuretics	0 (0.0)	0 (0.0)	0 (0.0)	(0.0)	1 (0.0)	0 (0.0)	1 (0.0)
trandolapril	1 (0.0)	1 (0.0)	4 (0.1)	7 (0.1)	1 (0.0)	3 (0.0)	3 (0.0)
trandolapril and diuretics	3 (0.0)	2 (0.0)	2 (0.0)	4 (0.1)	3 (0.0)	5 (0.1)	2 (0.0)
zofenopril	1 (0.0)	0 (0.0)	3 (0.0)	0 (0.0)	2 (0.0)	0 (0.0)	0 (0.0)
zofenopriland and diuretics	0 (0.0)	0 (0.0)	0 (0.0)	0 (0.0)	0 (0.0)	1 (0.0)	0 (0.0)

**Angiotensin II receptor blockers**							
losartan	15 (0.2)	19 (0.2)	12 (0.2)	13 (0.2)	13 (0.2)	10 (0.1)	12 (0.2)
losartan and diuretics	7 (0.1)	9 (0.1)	10 (0.1)	11 (0.1)	11 (0.2)	14 (0.2)	4 (0.1)
eprosartan	0 (0.0)	0 (0.0)	1 (0.0)	0 (0.0)	0 (0.0)	0 (0.0)	0 (0.0)
valsartan	16 (0.2)	19 (0.2)	7 (0.1)	9 (0.1)	12 (0.2)	4 (0.1)	6 (0.1)
valsartan and diuretics	14 (0.2)	14 (0.2)	17 (0.2)	19 (0.3)	15 (0.2)	9 (0.1)	4 (0.1)
valsartan and amlodipine	9 (0.1)	25 (0.3)	24 (0.3)	17 (0.2)	25 (0.3)	20 (0.3)	16 (0.2)
irbesartan	28 (0.4)	24 (0.3)	29 (0.4)	21 (0.3)	20 (0.3)	26 (0.4)	19 (0.3)
irbesartan and diuretics	20 (0.3)	22 (0.3)	13 (0.2)	27 (0.4)	12 (0.2)	18 (0.2)	23 (0.3)
candesartan	16 (0.2)	17 (0.2)	15 (0.2)	16 (0.2)	21 (0.3)	22 (0.3)	21 (0.3)
candesartan and diuretics	3 (0.0)	12 (0.2)	14 (0.2)	7 (0.1)	10 (0.1)	7 (0.1)	5 (0.1)
telmisartan	7 (0.1)	8 (0.1)	9 (0.1)	4 (0.1)	7 (0.1)	12 (0.2)	8 (0.1)
telmisartan and diuretics	11 (0.1)	3 (0.0)	6 (0.1)	4 (0.1)	4 (0.1)	6 (0.1)	6 (0.1)
temisartan and amlodipine	2 (0.0)	1 (0.0)	4 (0.1)	3 (0.0)	0 (0.0)	2 (0.0)	2 (0.0)
olmesartan medoxomil	14 (0.2)	16 (0.2)	13 (0.2)	10 (0.1)	0 (0.0)	0 (0.0)	0 (0.0)
olmesartan meoxomilol and diuretics	7 (0.1)	8 (0.1)	8 (0.1)	2 (0.0)	0 (0.0)	0 (0.0)	0 (0.0)
olmesartan medoxomil and amlodipine	13 (0.2)	12 (0.2)	10 (0.1)	3 (0.0)	0 (0.0)	0 (0.0)	0 (0.0)
**Renin inhibitors**							
aliskiren	4 (0.1)	2 (0.0)	1 (0.0)	1 (0.0)	1 (0.0)	0 (0.0)	0 (0.0)
aliskiren et hydrochlorothiazide	3 (0.0)	0 (0.0)	1 (0.0)	0 (0.0)	0 (0.0)	0 (0.0)	0 (0.0)
**Contraceptives**	**2,931 (37.4)**	**3,325 (43.3)**	**3,395 (44.8)**	**3,372 (45.5)**	**3,089 (42.3)**	**3,244 (45.1)**	**2,959 (41.8)**
**Hormonal contraceptives for systemic use**							
norandhisterone and ethinylestradiol	9 (0.1)	6 (0.1)	7 (0.1)	2 (0.0)	1 (0.0)	(0.0)	(0.0)
norgestrel and and ethinylestradiol	17 (0.2)	11 (0.1)	8 (0.1)	1 (0.0)	(0.0)	(0.0)	(0.0)
levonorgestrel and ethinylestradiol	888 (11.3)	772 (10.1)	680 (9.0)	681 (9.2)	588 (8.0)	530 (7.4)	517 (7.3)
desogestrel and ethinylestradiol	0 (0.0)	1 (0.0)	0 (0.0)	0 (0.0)	0 (0.0)	0 (0.0)	0 (0.0)
gestoden and ethinylestradiol	0 (0.0)	0 (0.0)	0 (0.0)	0 (0.0)	1 (0.0)	0 (0.0)	0 (0.0)
etonogestrel	1,703 (21.7)	1,664 (21.7)	1,666 (22.0)	1,625 (21.9)	1,509 (20.6)	1,787 (24.8)	1,579 (22.3)
desogestrel and ethinylestradiol	(0.0)	656 (8.5)	860 (11.3)	892 (12.0)	848 (11.6)	782 (10.9)	747 (10.6)
**Emergency contraceptives**							
levonorgestrel (emergency)	218 (2.8)	121 (1.6)	77 (1.0)	61 (0.8)	55 (0.8)	92 (1.3)	72 (1.0)
ulipristal (emergency)	11 (0.1)	2 (0.0)	10 (0.1)	7 (0.1)	9 (0.1)	10 (0.1)	12 (0.2)
**Contraceptives for topical use**							
DIU with progestogen	116 (1.5)	126 (1.6)	126 (1.7)	137 (1.8)	125 (1.7)	144 (2.0)	110 (1.6)
**Sex hormones**	**2,663 (34.0)**	**2,311 (30.1)**	**2,114 (27.9)**	**1,688 (22.8)**	**1,575 (21.6)**	**1,350 (18.7)**	**1,139 (16.1)**
**Androgens**							
testosterone	2 (0.0)	3 (0.0)	2 (0.0)	1 (0.0)	1 (0.0)	1 (0.0)	2 (0.0)
androstanolone	0 (0.0)	1 (0.0)	4 (0.1)	1 (0.0)	0 (0.0)	0 (0.0)	0 (0.0)
**Estrogens (for systemic use)**							
estradiol	110 (1.4)	111 (1.4)	138 (1.8)	129 (1.7)	139 (1.9)	126 (1.7)	119 (1.7)
**Estrogens (for topical use)**							
estriol	549 (7.0)	499 (6.5)	409 (5.4)	177 (2.4)	137 (1.9)	76 (1.1)	60 (0.8)
promestrien	549 (7.0)	499 (6.5)	409 (5.4)	177 (2.4)	137 (1.9)	76 (1.1)	60 (0.8)
**Progestogens**							
medrogestone	0 (0.0)	1 (0.0)	1 (0.0)	1 (0.0)	0 (0.0)	0 (0.0)	1 (0.0)
nomegestrol	37 (0.5)	39 (0.5)	31 (0.4)	26 (0.4)	25 (0.3)	23 (0.3)	26 (0.4)
chlormadinone	19 (0.2)	19 (0.2)	20 (0.3)	16 (0.2)	25 (0.3)	21 (0.3)	14 (0.2)
promegestone	11 (0.1)	9 (0.1)	4 (0.1)	4 (0.1)	4 (0.1)	5 (0.1)	3 (0.0)
**Anti-androgen**							
cyproterone	8 (0.1)	10 (0.1)	9 (0.1)	11 (0.1)	10 (0.1)	8 (0.1)	3 (0.0)
cyproterone and estrogens	1 (0.0)	(0.0)	(0.0)	(0.0)	(0.0)	(0.0)	(0.0)
**Hormone replacement therapy**							
norandhisterone and esotrogens	2 (0.0)	1 (0.0)	1 (0.0)	(0.0)	(0.0)	(0.0)	(0.0)
progesterone and estrogens	1,775 (22.6)	1,483 (19.3)	1,354 (17.8)	1,160 (15.7)	1,075 (14.7)	919 (12.8)	756 (10.7)
medroxyprogesterone and estrogens	0 (0.0)	0 (0.0)	0 (0.0)	1 (0.0)	0 (0.0)	0 (0.0)	0 (0.0)
dydrogesterone and estrogens	2 (0.0)	1 (0.0)	1 (0.0)	0 (0.0)	2 (0.0)	1 (0.0)	0 (0.0)
norandhisterone and estrogens (sequential preparation)	0 (0.0)	0 (0.0)	0 (0.0)	0 (0.0)	1 (0.0)	0 (0.0)	0 (0.0)
medroxyprogesterone and estrogens (sequential preparation)	0 (0.0)	0 (0.0)	0 (0.0)	0 (0.0)	1 (0.0)	0 (0.0)	0 (0.0)
dydrogesterone and estrogens (sequential preparation)	4 (0.1)	4 (0.1)	3 (0.0)	2 (0.0)	4 (0.1)	2 (0.0)	3 (0.0)
**Gonadotropins and other ovulation stimulants**							
chorionic gonadotrophin	4 (0.1)	3 (0.0)	3 (0.0)	3 (0.0)	2 (0.0)	0 (0.0)	0 (0.0)
human menopausal gonadotrophin	7 (0.1)	4 (0.1)	7 (0.1)	13 (0.2)	10 (0.1)	12 (0.2)	12 (0.2)
urofollitropin	2 (0.0)	2 (0.0)	0 (0.0)	1 (0.0)	1 (0.0)	1 (0.0)	0 (0.0)
follitropin alfa	11 (0.1)	10 (0.1)	12 (0.2)	7 (0.1)	13 (0.2)	19 (0.3)	14 (0.2)
follitropin beta	5 (0.1)	3 (0.0)	5 (0.1)	2 (0.0)	2 (0.0)	7 (0.1)	3 (0.0)
lutropin alfa	0 (0.0)	0 (0.0)	0 (0.0)	0 (0.0)	0 (0.0)	0 (0.0)	1 (0.0)
choriogonadotropin alfa	12 (0.2)	14 (0.2)	15 (0.2)	15 (0.2)	8 (0.1)	18 (0.2)	18 (0.3)
clomifene	33 (0.4)	56 (0.7)	45 (0.6)	49 (0.7)	48 (0.7)	49 (0.7)	45 (0.6)
**Antigonadotropins and similar agents**							
danazol	2 (0.0)	0 (0.0)	0 (0.0)	2 (0.0)	2 (0.0)	1 (0.0)	0 (0.0)
**Sex homones for systemic disease**							
raloxifen	1 (0.0)	0 (0.0)	0 (0.0)	1 (0.0)	2 (0.0)	1 (0.0)	0 (0.0)
ulipristal (ESMYA)	0 (0.0)	3 (0.0)	5 (0.1)	7 (0.1)	3 (0.0)	2 (0.0)	3 (0.0)
