# Supplementary material for: Trends in harmful drug exposure during pregnancy in France between 2013 and 2019: A nationwide cohort study
Source: PLoS One. 2024 Jan 10;19(1):e0295897. doi: 10.1371/journal.pone.0295897 (PMC10781191; doi:10.1371/journal.pone.0295897)
Supplement: S8 Table — (PDF) [file pone.0295897.s008.pdf]

S8 Table: Maternal and pregnancy outcomes characteristics (sensitivity analysis without topical retinoids and topical NSAIDs)

	Overall<br>n=5,253,284	Pregnancies not exposed to a harmful drug<br>n=5,048,882	Pregnancies exposed to at least one harmful drug<br>n=128,085
**Prevalence over whole cohort**	-	96.1%	2.4%
**Pregnant women**	4,074,996	3,952,839	124,175
**Maternal age (years)**			
**Mean (+/- SD)**	30.3 +/- 5.4	30.2 +/- 5.4	30.5 +/- 5.9
**< 20**	110,055 (2.1%)	104,448 (2.1%)	3,871 (3.0%)
**20-29**	2,247,780 (42.8%)	2,161,430 (42.8%)	51,837 (40.5%)
**30-39**	2,659,845 (50.6%)	2,559,672 (50.7%)	64,105 (50.1%)
**≥ 40**	235,604 (4.5%)	223,332 (4.4%)	8,272 (6.5%)
**Chronic disease in the year prior or during pregnancy**			
**Psychiatric troubles**	212,278 (4.0%)	196,074 (3.9%)	12,125 (9.5%)
**Pre-gestational diabetes**	36,270 (0.7%)	32,702 (0.7%)	2,720 (2.1%)
**Hypertension**	74,276 (1.4%)	66,200 (1.3%)	6,420 (5.0%)
**Number of hospitalisations in the year prior to pregnancy**			
**Mean (+/- SD)**	0.3 +/- 1.2	0.3 +/- 1.2	0.5 +/- 1.5
**none**	4,033,101 (76.8%)	3,887,928 (77.0%)	89,137 (69.6%)
**1**	883,643 (16.8%)	843,059 (16.7%)	26,294 (20.5%)
**2 or more**	336,540 (6.4%)	317,895 (6.3%)	12,654 (9.9%)
**Low-income status***	240,564 (4.6%)	224,659 (4.5%)	10,010 (7.8%)
**Pregnancy outcomes**			
**Live births**	5,129,561 (97.6%)	4,929,552 (97.6%)	124,735 (97.4%)
**Medical termination <22GW**	79,071 (1.5%)	76,606 (1.5%)	1,964 (1.5%)
**Medical termination≥22GW**	18,274 (0.4%)	17,541 (0.4%)	555 (0.4%)
**Still births**	26,378 (0.5%)	25,183 (0.5%)	831 (0.7%)
**Gestational age at birth (for live births only)**			
**Mean (+/- SD)**	39.0 +/- 1.9	39.0 +/- 1.9	38.9 +/- 1.9
**Premature birth < 37GW**	323,042 (6.2%)	309,754 (6.1%)	8,666 (6.8%)
**<28GW**	15,848 (0.3%)	15,259 (0.3%)	428 (0.3%)
**[28-31] GW**	30,236 (0.6%)	29,020 (0.6%)	823 (0.6%)
**[32-36] GW**	276,958 (5.3%)	265,475 (5.3%)	7,415 (5.8%)

*****Low-income status was defined as affiliation to CMUc

Abbreviations: CMU (*couverture maladie universelle*), GW (gestational week), SD (standard deviation)

Data are shown as mean (+/- SD) or n (%)
