# Supplementary material for: Trends in harmful drug exposure during pregnancy in France between 2013 and 2019: A nationwide cohort study
Source: PLoS One. 2024 Jan 10;19(1):e0295897. doi: 10.1371/journal.pone.0295897 (PMC10781191; doi:10.1371/journal.pone.0295897)
Supplement: S9 Table — (PDF) [file pone.0295897.s009.pdf]

S9 Table: Maternal and pregnancy outcome characteristics among pregnancies exposed to at least one teratogenic drug (sensitivity analysis without topical retinoids)

	During whole pregnancy n=5,253,284	During preconceptional period or T1 n=5,253,284	During preconceptional period n=5,253,284	During T1 n=5,253,284	During T2 n=5,210,429	During T3 n=5,149,745
**Exposed pregnancies to at least one teratogenic drug, n (%)**	27,930 (0.5%)	25,671 (0.5%)	22,412 (0.4%)	11,767 (0.2%)	5,128 (0.1%)	4,264 (0.1%)
**Pregnant women**	26,472	24,349	21,274	11,203	4,788	3,939
**Maternal age (years)**						
**Mean (+/- SD)**	32.1 +/- 5.5	32.1 +/- 5.5	32.1 +/- 5.5	32.5 +/- 5.6	32.2 +/- 5.7	32 +/- 5.4
**< 20**	221 (0.8%)	190 (0.7%)	154 (0.7%)	99 (0.8%)	57 (1.1%)	32 (0.8%)
**20-29**	8,990 (32.2%)	8,195 (31.9%)	7,112 (31.7%)	3,535 (30.0%)	1,611 (31.4%)	1,394 (32.7%)
**30-39**	16,131 (57.8%)	14,871 (57.9%)	13,053 (58.2%)	6,831 (58.1%)	2,954 (57.6%)	2,486 (58.3%)
**≥ 40**	2,588 (9.3%)	2,415 (9.4%)	2,093 (9.3%)	1,302 (11.1%)	506 (9.9%)	352 (8.3%)
**Chronic disease in the year prior or during pregnancy**						
**Psychiatric troubles**	6,031 (21.6%)	5,786 (22.5%)	5,304 (23.7%)	2,864 (24.3%)	1,069 (20.9%)	999 (23.4%)
**Pre-gestational diabetes**	1,621 (5.8%)	1,568 (6.1%)	1,415 (6.3%)	776 (6.6%)	220 (4.3%)	102 (2.4%)
**Hypertension**	3,527 (12.6%)	3,415 (13.3%)	3,163 (14.1%)	1,630 (13.9%)	492 (9.6%)	317 (7.4%)
**Number of hospitalisations in the year prior to pregnancy**						
**Mean (+/- SD)**	0.7 +/- 2.5	0.7 +/- 2.4	0.7 +/- 2.3	0.7 +/- 2.7	0.7 +/- 3.6	0.6 +/- 1.5
**none**	17,930 (64.2%)	16,297 (63.5%)	14,087 (62.9%)	7,488 (63.6%)	3,432 (66.9%)	2,914 (68.3%)
**1**	6,006 (21.5%)	5,598 (21.8%)	4,927 (22.0%)	2,571 (21.9%)	1,064 (20.8%)	854 (20.0%)
**2 or more**	3,994 (14.3%)	3,776 (14.7%)	3,398 (15.2%)	1,708 (14.5%)	632 (12.3%)	496 (11.6%)
**Low-income status***	1,615 (5.8%)	1,460 (5.7%)	1,222 (5.5%)	698 (5.9%)	290 (5.7%)	216 (5.1%)
**Pregnancy outcome**						
**Live births**	26,696 (95.6%)	24,474 (95.3%)	21,340 (95.2%)	11,119 (94.5%)	4,975 (97.0%)	4,233 (99.3%)
**Medical termination <22GW**	806 (2.9%)	799 (3.1%)	717 (3.2%)	440 (3.7%)	41 (0.8%)	(0.0%)
**Medical termination≥22GW**	167 (0.6%)	160 (0.6%)	143 (0.6%)	89 (0.8%)	47 (0.9%)	12 (0.3%)
**Still births**	261 (0.9%)	238 (0.9%)	212 (1.0%)	119 (1.0%)	65 (1.3%)	19 (0.5%)
**Gestational age at birth (for live births only)**						
**Mean (+/- SD)**	38.6 +/- 2.3	38.6 +/- 2.3	38.5 +/- 2.3	38.5 +/- 2.4	38.5 +/- 2.3	38.9 +/- 1.7
**Premature birth < 37GW**	2,891 (10.4%)	2,699 (10.5%)	2,380 (10.6%)	1,293 (11.0%)	578 (11.3%)	323 (7.6%)
**<28GW**	172 (0.6%)	163 (0.6%)	143 (0.6%)	87 (0.7%)	37 (0.7%)	(0.0%)
**[28-31] GW**	345 (1.2%)	329 (1.3%)	292 (1.3%)	163 (1.4%)	65 (1.3%)	14 (0.3%)
**[32-36] GW**	2,374 (8.5%)	2,207 (8.6%)	1,945 (8.7%)	1,043 (8.9%)	476 (9.3%)	309 (7.3%)

*Low income status was defined as affiliation to CMUc

Abbreviations: CMU (*couverture maladie universelle*), GW (gestational week), SD (standard deviation)

Data are shown as mean (+/- SD) or n (%)
