# Supplementary material for: Trends in harmful drug exposure during pregnancy in France between 2013 and 2019: A nationwide cohort study
Source: PLoS One. 2024 Jan 10;19(1):e0295897. doi: 10.1371/journal.pone.0295897 (PMC10781191; doi:10.1371/journal.pone.0295897)
Supplement: S10 Table — (PDF) [file pone.0295897.s010.pdf]

S10 Table: Maternal and pregnancy outcome characteristics among pregnancies exposed to at least one fetotoxic drug (sensitivity analysis without topical NSAIDs)

	During T2 or T3 n=5,210,429	During T2 n=5,210,429	During T3 n=5,149,745
**Exposed pregnancies to at least one fetotoxic drug, n (%)**	101,619 (2.0%)	57,371 (1.1%)	48,002 (0.9%)
**Pregnant women**	99,416	56,557	47,207
**Maternal age (years)**			
**Mean (+/- SD)**	30.1 +/- 5.9	30.3 +/- 6	29.9 +/- 5.8
**< 20**	3,662 (3.6%)	1,954 (3.4%)	1,909 (4.0%)
**20-29**	43,279 (42.6%)	23,876 (41.6%)	20,825 (43.4%)
**30-39**	48,803 (48.0%)	27,719 (48.3%)	22,916 (47.7%)
**≥ 40**	5,875 (5.8%)	3,822 (6.7%)	2,352 (4.9%)
**Chronic disease in the year prior or during pregnancy**			
**Psychiatric troubles**	6,386 (6.3%)	3,763 (6.6%)	2,969 (6.2%)
**Pre-gestational diabetes**	1,254 (1.2%)	863 (1.5%)	479 (1.0%)
**Hypertension**	3,218 (3.2%)	2,262 (3.9%)	1,295 (2.7%)
**Number of hospitalisations in the year prior to pregnancy**			
**Mean (+/- SD)**	0.4 +/- 1	0.5 +/- 1.1	0.4 +/- 0.9
**none, n (%)**	72,115 (71.0%)	40,199 (70.1%)	34,491 (71.9%)
**1**	20,629 (20.3%)	11,927 (20.8%)	9,514 (19.8%)
**2 or more**	8,875 (8.7%)	5,245 (9.1%)	3,997 (8.3%)
**Low-income status***	8,533 (8.4%)	5,262 (9.2%)	3,714 (7.7%)
**Pregnancies issues**			
**Live births**	99,452 (97.9%)	55,381 (96.5%)	47,790 (99.6%)
**Medical termination <22GW**	1,180 (1.2%)	1,180 (2.1%)	(0.0%)
**Medical termination≥22GW**	397 (0.4%)	348 (0.6%)	53 (0.1%)
**Still births**	590 (0.6%)	462 (0.8%)	159 (0.3%)
**Gestational age at birth for live births**			
**Mean (+/- SD)**	39 +/- 1.8	38.8 +/- 2	39.2 +/- 1.4
**Premature birth < 37GW**	5,920 (5.8%)	4,442 (7.7%)	1,698 (3.5%)
**<28GW**	267 (0.3%)	267 (0.5%)	0 (0.0%)
**[28-31] GW**	493 (0.5%)	449 (0.8%)	53 (0.1%)
**[32-36] GW**	5,160 (5.1%)	3,726 (6.5%)	1,645 (3.4%)

*Low income status was defined as affiliation to CMUc

Abbreviations: CMU (*couverture maladie universelle*), GW (gestational week), SD (standard deviation)

Data are shown as mean (+/- SD) or n (%)
