# Supplementary figures and images for: Trends in harmful drug exposure during pregnancy in France between 2013 and 2019: A nationwide cohort study
Source: PLoS One. 2024 Jan 10;19(1):e0295897. doi: 10.1371/journal.pone.0295897 (PMC10781191; doi:10.1371/journal.pone.0295897)

**S1 Figure:** Flow chart.

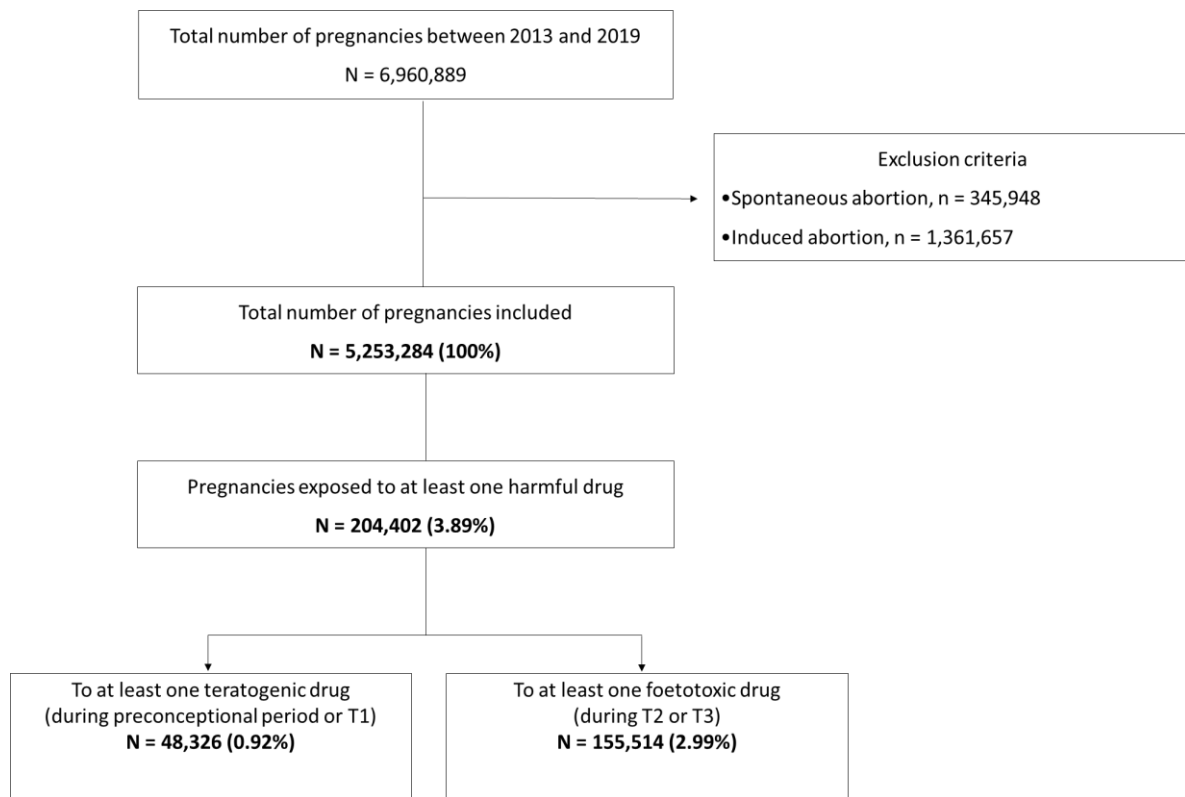

Supplement: S1 Fig — (PDF) [file pone.0295897.s011.pdf]
