# Supplementary material for: Trends in harmful drug exposure during pregnancy in France between 2013 and 2019: A nationwide cohort study
Source: PLoS One. 2024 Jan 10;19(1):e0295897. doi: 10.1371/journal.pone.0295897 (PMC10781191; doi:10.1371/journal.pone.0295897)
Supplement: Attachment — Submitted filename: Response to Reviewers.docx [file pone.0295897.s012.docx]

|  | **Response to Reviewers** | **Mention in the manuscript** |
| --- | --- | --- |
|  | | |
| **Editor’s response** | | |
| Thank you for submitting your manuscript to PLOS ONE. After careful consideration, we feel that it has merit but does not fully meet PLOS ONE’s publication criteria as it currently stands. Therefore, we invite you to submit a revised version of the manuscript that addresses the points raised during the review process. | Thank you for your attention and for your response. We are pleased to respond to each point raised by the reviewer | *Not available* |
| Please add, recent years in your analysis if possible | We added one extra year to our analysis (2019) in addition to previous analysis. We decided to not add the year 2020 because of Covid pandemic as access to care (medical consultations and drug consumption) were indeed particular and did not reflect usual drug exposure.  Furthermore, we identified a mistake in the original manuscript. Hence, we described in the year “2012” pregnancies related to a date of conception during the year 2012. However, our database starts from January 2012. As a consequence, preconceptional information for pregnancies that have begun from January to March 2012 (preconceptional period in 2011) was not available. To address this issue, we decided to start study period in January 2013. This change also allows to ensure comparability between complete calendar years (12-months for each year in the study) | *Not available* |
| Please provide a graphical abstract in eSupplement | We added a graphical abstract in addition to the submission. | *Not available* |
| Please be sure than all assumptions discussed in the appropriate section are based on your own results. | We reviewed all assumptions discussed in the manuscript according to our results. | Discussion has been revised accordingly |
|  |  |  |
| **Comments to the Author: General comments** |  |  |
|  |  |  |
| **Review Comments to the Author** | | |
| **General comments** | | |
| This research has public health implications for France and for other countries that monitor the medicines used in pregnancy. Similar studies have been published recently indicating a high use of harmful medicines including teratogenic medicines. This study has the potential to complement the existing data on medicine use in pregnancy in France. The current study should also attempt to describe why the findings of this study are different from the other studies. | We did our best to underline why our findings complement previous studies. First, we studied harmful drugs according to the type of risk for the fetus and according to the critical period of exposure (preconceptional or T1 for teratogenic drugs; T2 or T3 for foetotoxic drugs). Furthermore, these exposures were analyzed according to year of pregnancy, in order to provide trends overs years in a recent period. | *Page 13, lignes 199-204*  *Page 14, lignes 221-226*  *Page 21, lignes 396-402* |
| The study presented prevalence of medicine use in pregnancy, specifically in the three months pre-pregnancy and three trimesters of pregnancy. This is a very useful presentation of results to understand medicine use in pregnancy, and exposure of specific pregnancy related periods which have different susceptibility to foetal and embryonic harms. The medicines were categorized as teratogenic and fetotoxic, based on the pregnancy-related period when these medicines are deemed harmful. However, data was not presented for all the pregnancy-related time periods for fetotoxic medicines. | Our study is a description of harmful drug exposure in accordance with specific period (when exposure has clinical, teratogenic or fetotoxic, impact). Fetotoxic drugs as NSAIDs have no impact and are not contra-indicated during the preconceptional period nor the first trimester. In accordance, we did not study those periods for fetotoxic drugs. On the contrary, teratogenic drugs are contra-indicated during the whole pregnancy as some organ/tissues may be affected after T1. We provide these results related to exposure during the four periods (from preconceptional period to birth) in supplemental data. | *Supporting Information,*  *Page 5, S3 Table* |
| The study found that topical retinoids and systemic NSAIDs were the most used harmful medicines in pregnancy in France between 2012 and 2018. The authors note that topical retinoids are not considered as harmful as the systemic retinoids, but that these are contraindicated for use in pregnancy, by the regulatory authorities. I feel the study would benefit from a sensitivity analysis to demonstrate prevalence rates with and without retinoids in the mix. | We thank the reviewer for this relevant point. Although they diffuse into systemic circulation, the absorbed dose is lower for topical agents than after oral administration; and the risk is probably lower.  As suggested, we performed three sensitivity analyses excluding topical retinoids, topical NSAIDs, and both.  Here are the results of these sensitivity analyses:   - Whole pregnancy without topical retinoids and topical NSAIDs: 2.44% of pregnancies were exposed to at least one harmful drug (*vs.* 3.89% in the original analysis). - During preconceptional period and T1 without topical retinoids: 0.49% of pregnancies were exposed to at least one teratogenic drug (*vs.* 0.92%). - During T2 or T3 without topical NSAIDs: 1.95% of pregnancies were exposed to at least one fetotoxic drug (*vs.* 2.99%). | *Page 19, lignes 356-363* |
| In my opinion, the study results should be reported in a positive light, that other more harmful systemic medicines were rarely used in France in the time period considered. Also, a thorough spell check, language check and rephrasing of several paragraphs is recommended. | We thank the reviewer for this comment. We modified some expression (mostly in abstract and conclusion) as overall exposure is rare considering all the pregnancies at a national level. There is indeed a decrease, although persistent, exposure to harmful drug during sensitive periods. | *Page 3, lignes 52-53*  *Page 26, lignes 505-506* |
|  |  |  |
| **Introduction:** |  |  |
| The section needs a thorough rewriting in light of the aims of the study. It is not clear what each paragraph aims to do. I have provided some big picture feedback below: 1. The following statements should be rephrased to describe what is intended from this paragraph: “Most previous studies evaluate prevalence of potentially harmful drug using the FDA risk classification system. But since 2015, it has been abandoned and replaced by a narrative description of fetal risks of drugs (9).” I think the authors want to describe the inconsistencies in the prevalence of medicine use in pregnancy, because of the different classification systems used. It is not clear why the authors point out that the FDA’s alphabetic classification system is no longer in use- what would the authors like to imply from this sentence? This paragraph needs further work- it specifically needs clarifying as to what the aim is: It seems like the authors intend to compare French utilization rates with other developed countries and describe the reasons for differences. However, this is not clear from the paragraph. | We rephrased some paragraph and clarified how the use of different classification system may have led to publish different results on potentially harmful drug prevalence.  In the manuscript, we have mentioned FDA classification because most previous studies were based on, however explaining that it is no longer use. We mentionned FDA classification to introduce the lack of classification for harmful drugs during pregnancy in France. We decided to erase a short paragraph which described previous old and foreign studies. | *Page 4, lignes 69-71* |
| 2. The final paragraph should summarise the gaps in the published French literature. This paragraph only points out one study that reported 2.4% of pregnancies exposed to NSAIDs. I believe other published French studies on medicines used in pregnancy have used some form of risk classification system. This paragraph or the one before should point this out clearly, and the resulting prevalence estimates as well, for the reader to make sense of what exists, and what gaps are being fulfilled by this study. | We also, as suggested, clarified the final paragraph of the introduction with underlying what was missing in the current literature. Previous studies described prevalence of specific drug class such as antiepileptics and NSAIDs. We reported them in the introduction. However, no study described trends over the last 10 years, and we believe that point is importance in the goal of public policies for pregnant women. | *Page 5, lignes 84-86* |
|  |  |  |
| **Methods** |  |  |
| The study included pregnancies with date of conception between 2012 and Dec 2018, meaning that some pregnancies resulted in a birth in 2019 as well. Perhaps the authors should revisit how the data is presented. Because maternal characteristics and use is described during pregnancy, the data on utilization (including graphs) should account for date of the birth instead of the date of conception, for a better representation of data. This is relevant because the prescriptions would have been filled in 2019, not 2018, for women who conceived in December 2018. | Thank you for underlying this issue. In the manuscript, we choosed to use the date of conception for the year of exposure because date of conception appears more relevant. As you mentioned, foetotoxic drugs are used in the 3^rd^ trimester and the date of use is closed to the date of birth. However, for teratogenic drugs exposed in preconceptional period and T1, date of exposure is closer to date of conception. Furthermore, other studies using the same database consider the year of exposure regarding to the beginning of pregnancy (1,2). We do not think that this could have major impact on data because changes over years are smooth. | *Page 15, ligne 268*  *Page 16, ligne 284*  *Pages 17, 18, 19* |
|  |  |  |
| **Results:** |  |  |
| Table1: This table is very informative with details of the cohort and has implications for generalizability. The abbreviations need explanatory footnotes.  In my opinion, Table S4 is more informative for the results section because it describes use by specific trimesters. Could the authors amend table 1 to reflect trimester-wise use? In the introductory paragraphs, the authors claim that no other French study provides data by trimester. This table might fulfill that gap- and it is relevant to fetotoxic medicines as well. | As you mentioned, we added explanatory footnotes for the abbreviations to amend Table 1.  We also added Tables 2 and 3 to describe population characteristics for each case of drug exposure (related to risk and pregnancy period). So that, we described pregnancy characteristics in case of exposure to teratogenic drugs and in case of exposure to fetotoxic drugs. Population data is yet more exhaustive. | *Table 1, page 10*  *Table 2, page 11*  *Table 3, page 12* |
| Figure1: Contraceptive use in T3 is very high. The discussion does not really go into the details of why some medicines or medicine groups were more prevalent in one trimester but not the other. Secondly, regarding contraceptives, why did its prevalence not show in pre-pregnancy period? I would have expected to see high peaks of Contraceptives in pre-pregnancy and T1. In T1 I assume women receive a dispensing until they become aware of the pregnancy status. Likewise, for sex hormones (includes fertility medicines). I see that pre-pregnancy and T1 use was not described for Fetotoxic medicines which are contraindicated in later trimesters. Even so, it is good to present this data especially for international comparisons, given that these medicines are mostly categorised teratogenic using pregnancy risk categorisation systems. | Regarding contraceptives, we underlined that use in T3 was higher than in T2. We developed this change in discussion and supposed that women buy contraception before delivery and used it in the *post-partum* period. Our database, based on reimbursement, cannot evaluate real drug intake. However, we did not evaluate contraceptives prevalence in preconceptional nor in T1 because we focus on pharmacological risks which depend on period of exposure. | *Page 14, lignes 224-226*  *Page 24, lignes 487-489* |
| Psychiatric disorders were found in 2% pregnancies, and 12% in women exposed to teratogenic meds.- how does that compare with antiepileptic use and psychiatric medicine use? I feel its worth commenting in the discussion section. | Thank you for your comment. We developed an additional paragraph named “Patterns of exposed mothers” in the discussion. | *Page 20* |
|  |  |  |
| **Discussion** |  |  |
| 1. I think the discussion needs more argument and placing the study in existing literature. Recent French studies have found much higher prevalence of teratogenic medicine use. What are the probable reasons that this study found such low rates? It is worth comparing these estimates with the present study. Having said that the discussion also needs a paragraph on what this study adds to existing data on medicine use in pregnancy in France. As the authors have pointed out, a few studies have reported use of harmful medicines in pregnancy in recent years. How does the current study complement those results? | Recent French studies that have found much higher prevalence of harmful medicine use did not focus on specific drugs (as NSAIDs) nor specific route of administration but on larger categories (as anti-inflammatory and antirheumatic products) including systemic and topical uses.  We add some comments in the discussion paragraph to underline how our study is in accordance and complements previous studies. | *Page 23; lignes 449-452* |
| 2. While the authors discuss trends of use of broad medicine groups in the discussion on temporal trends through pregnancy and through the calendar years, specific medicines are not discussed. For example, the figures show Lithium use has increased- what are its implications? | We thank the reviewer for this comment. We definitely agree that it would be interesting to study other drugs, such as lithium. However, the main purpose of this study was to describe trends overs years and we choose to focus on the major drugs, in order to provide data for health authorities. We plan to assess specific exposure and their determinants in another ongoing study. | *Not available* |
|  |  |  |
| **Conclusion** |  |  |
| Conclusions may be described differently- Firstly, the results do not indicate decreasing exposures in sensitive periods (T1 presumably). However, the conclusions indicate so. Secondly, the paper claims that the results indicate the importance of keeping healthcare professionals and patients informed. This aspect does not shine through in the methods and results. Were there ongoing interventions in France that aimed to increase the understanding of teratogenicity and fetotoxicity? If so, this should be addressed in the discussion. | We agree that the conclusion was not fully appropriate according to our findings. It has been reworded to better reflect our results and main messages of this study. | *Page 26* |

1.	Blotière PO, Weill A, Dalichampt M, Billionnet C, Mezzarobba M, Raguideau F, et al. Development of an algorithm to identify pregnancy episodes and related outcomes in health care claims databases: An application to antiepileptic drug use in 4.9 million pregnant women in France. Pharmacoepidemiol Drug Saf. 2018;27(7):763–70.

2.	Blotière PO, Damase-Michel C, Weill A, Maura G. Dispensing of Potentially Harmful Prescription Drugs in 1.8 Million Pregnant Women in France: A Nationwide Study Based on Two Risk Classification Systems. Drug Saf. 2021 Oct 6;
