# Supplementary material for: Trends in harmful drug exposure during pregnancy in France between 2013 and 2019: A nationwide cohort study
Source: PLoS One. 2024 Jan 10;19(1):e0295897. doi: 10.1371/journal.pone.0295897 (PMC10781191; doi:10.1371/journal.pone.0295897)
Supplement: Attachment — Submitted filename: Response to Reviewers.docx [file pone.0295897.s013.docx]

|  | **Response to Reviewers** | **Mention in the manuscript** |
| --- | --- | --- |
|  | | |
| **Overall Comments** | | |
| The authors have made changes to the manuscript, but it still needs significant work | Thank you for your attention and for your comments. We are pleased to respond to each point. | *Not available* |
| The writing is not up to the standards of PlosOne. The language used is inconsistent and needs a lot of work. | We did our best to rewrite the manuscript with better language and rephrase some paragraphs. | *Not available* |
|  |  |  |
| **Abstract** | | |
| Methods: How long was the preconceptional period. An abstract is a standalone piece. Its more informative to a reader if you have described it up front, as the authors have done for trimester duration | We added an extra explanation in the abstract: “Each pregnancy was divided into a preconceptional period of 90 days period before conception and three trimesters from conception to birth”. | *Page 2, lignes 40-41* |
| Outcome: Drug exposure might need further description: Percents? No. of women exposed? No. of babies? Pregnancies? | Our study is a description of prevalence of pregnancies using at least one harmful drug during the critical period of interest. In this study, the unit of analysis is the pregnancy, and each woman can account for more than one pregnancy during study period. We rephrased the sentence. | *Page 2, ligne 45* |
| Results: Needs rewriting with better language, and careful use of tenses:   - For e.g. “One over 23 pregnant women”? Do the authors mean 1 in 23- i.e. about a quarter? | This sentence referred to the use of harmful drug in 3.89% of pregnancies. We used a different expression of this finding to better fits with physician expectation in their routine activities. Hence, here, “about one over 25 pregnancies “ | *Page 3, ligne 57* |
| - Exposure decreases’- perhaps change to ‘exposure to teratogenic and fetotoxic meds decreased from preconception through TO birth…. Something on these lines. | We thank the reviewer to help us clarifying our results. We better described the period of interest, which corresponds in this sentence to the period ranging from the preconceptional period to the end of pregnancy. | *Page 3, lignes 52-54* |
| - Also, this is the first time the authors describe a ‘critical period’. This needs a mention in methods too. | We defined more precisely the critical period: it referred to “preconceptional period or first trimester for teratogenic drugs and second or third trimesters for fetotoxic drugs.” | *Page 2, lignes 43-44* |
| - Could the author ensure the difference between ‘use’ and ‘exposure’ from the iuyset? Because this is a database analysis, ‘use’ is not automatically presumed. Even so, if using ‘exposure’ please stick to that, or choose ‘use’ and describe in the main text that use may not be presumed but the term in the manuscript implies exposure. | We thank the reviewer for this relevant point. We corrected the language in the manuscript by choosing the word “exposure” and defined it in the method section (i.e corresponding to drug dispensing). Additionally, to be in accordance with the all manuscript, we changed “use” into “exposure” in the title. | *Not available* |
| Conclusion: Language edits needed; unnecessary use of words like ‘providing a comprehensive analysis’- word space could be better utilised for further drawing conclusions | Thank you for underlying this issue. We did our best to rewrite the manuscript with better English language. | *Not available* |
|  |  |  |
| **Introduction:** |  |  |
| The authors must aim to have a professional edit done for language and terminologies use.  E.g. I am not sure if ‘sanitary disasters’ is a commonly used term for the thalidomide tragedy, and ‘dramatic injuries’ for foetal/congenital malformations? Also the wording can be more concise and succinct. | We rewrote the sentence with other words. | *Page 4, lignes 64-66* |
| The authors claim the FDA classification was abandoned in 2015 (Line 70)- this needs revisiting. I am aware the FDA classification became descriptive, in place of the alphabetic (A,B,C,D,X)- this fact is not forthcoming. What do the authors mean by clinical teratologist assessment in view of the classification? There are no references either? | We described the alphabetic FDA classification because it is still sometimes presented as such, even in recent publication. We acknowledge the absence of a harmful drug list available at a national level in France. In this study, harmful drugs were defined using existing classification elsewhere and based on our clinical and pharmacological expertise. | *Page 4, ligne 86*  *Page 5, lignes 87-90* |
| Line 72: “In developed countries members of the OCDE, prescription of potentially harmful drug”….” What is OCDE? OECD? Please define at first use. | Thank you for underlying this abbreviation. It referred to the Organization for Economic Cooperation and Development (OECD). | *Page 4, lignes 85-86* |
| Because the author refer to FDA classification, perhaps describe what category X here is? Not all readers are aware how risk categories work? | We rephrased this paragraph with additional explanation. | *Page 4, ligne 86*  *Page 5, lignes 87-90* |
| Line 77-78: “Although prevalence of women who received a prescription of potentially harmful drug differed based upon the sources used…” What do the authors mean by sources used- do they mean pregnancy risk classification systems? | We rephrased this paragraph. Variations in the prevalence of potentially harmful drug prescriptions may be explained by the use of different source of data (mostly claim databases and questionnaires) and different risk classifications. | *Page 4, lignes 82-83* |
| Line 87: “Considering Non-Steroidal Anti-Inflammatory Drugs (NSAIDs), 2.3% of pregnancies were exposed after the sixth month of gestation….” – Is there a reason these medicines were highlighted in the introduction? Are these the most commonly used? I can see the point is using valproic acid and antiepileptic- even though it needs better argument here in the introduction- their potential to cause damage to foetus! The authors should highlight this to make their point, which will flow through to their results well later on. | We rephrased this paragraph to better explain why those drug categories are highlighted in the introduction. NSAIDs are the most prescribed harmful drugs. | *Page 5, lignes 94-95* |
| In line 90 the authors mention “This pharmacological class was the most prescribed among the harmful drugs.”- when? In the study reported in the previous line or in the current paper? If it’s the latter, this sentence must be deleted because the introduction should not report results of the paper. If it’s the former, the sentences need to be merged using better language and argument. | We rephrased the sentence to improve the paragraph understanding. | *Page 5, lignes 94-95* |
| Line 91: “To date, no study has been performed to assess the exposure during pregnancy specifically according to the type of consequence for the fetus”- It is not clear what the authors are aiming for. What do they mean by ‘type of consequence’? Is that safety outcomes like foetal malformation?? Or clinical effectiveness? | In this sentence we referred to the teratogenic or fetotoxic risk for the fetus. We rephrased the sentence. | *Page 5, lignes 100-101* |
| Line 94: ‘critical period of exposure’ Again the authors do not describe what this means? This is the first time the term has been used- this should have been described in the prior paragraphs where international data was described. Also it is important to go into the detail of ‘critical’ period- what is it and why is it critical. | In abstract and introduction, we defined more precisely the critical period. It referred to the period during which each drug is considered harmful: preconceptional period or first trimester for teratogenic drugs and second and third trimesters for fetotoxic drugs. | *Page 4, lignes 69-75* |
| What was the rationale of presenting the trends of exposure in this study? This rationale needs better development. | Recent therapeutic advances have changed the management of certain chronic disease, including in pregnancy (eg increased used of TNF alpha blockers for inflammatory arthritis). Besides valproate issue was highly broadcasted in media years ago, and lead to communications on the safety of drug during pregnancy, and we hope some awareness of physician and women. Hence, it appeared important to describe trends of use over the recent years of harmful drugs during pregnancy and to provide data for settle public health communication and policies. | *Page 5, ligne 102* |
|  |  |  |
| **Methods** |  |  |
| Line 103: “costly long term disease codes”- what do the authors mean? Does the SNDS include diagnosis codes of chronic diseases And how do they define ‘costly’- are these diseases costly to treat? Perhaps get rid of this term? | We referred here to codes used by the national health insurance for chronic diseases. We erased the useless mention to health cost. | *Page 6, ligne 109* |
| Line 104-105: “corresponding to full reimbursement for low income”- does the data only have reimbursement for low income, or is that the only population for which the healthcare is reimbursed? | The SNDS collects all the health data associated with a health insurance reimbursement whether it is fully or partly reimbursed. CMUc corresponds to a public supplementary health coverage that allows to get reimbursed the additional part of health expenses. It thus corresponds to free access to health care. CMUc affiliation (Couverture Maladie Universelle complémentaire) is allowed for people with low income. | *Page 6, lignes 110-111* |
| It is not clear how the date of conception was estimated using the date of the last menstrual period or the gestational age- how many days/months prior? What was the calculation used: this is important because it will provide an insight into the estimated exposure timing. | We rephrased the explanation. Date of conception was calculated using 2 variables encoded in the hospital discharge database at birth: the date of pregnancy outcome and the numbers of days since the last menstrual period. Date of conception is estimated to be fourteen days after the date of last menstrual period. | *Page 7, lignes 126-130* |
| Date of conception was used to define pregnancy: this should be stated upfront. | Thank you for your advice. We corrected redaction in the manuscript. | *Page 6, ligne 121* |
| Study provides exposure data on pregnancies between 2013 and 2020, as opposed to what authors claim: 2013 to 2019: here I would like to highlight ‘2020’ given that some pregnancies with conception date in Dec 2019 would end in a birth or would exceed the 22-week duration in the following year. Therefore, I hope the authors have cross checked the exposure dates in their datasets when they reported the results? | We included pregnancies for which the date of conception ranged from January 2013 to December 2019. Each pregnancy has been followed until pregnancy outcome. They may thus be exposed in 2020. However, as commonly performed in drug utilization study during pregnancy, date refers to the date of conception for each pregnancy, not to the actual date of exposure. | *Not available* |
|  |  |  |
| **Results:** |  |  |
| Given that the authors only report data for T1 and preconception for teratogenic meds, and for T2-T3 for fetotoxic meds, perhaps best to provide two separate graphs within the same figure. At the moment it looks like the cohort just never received NSAIDs for example. | We thank the reviewer for highlighting this issue. We draw two separate graphs for the figure to better separate teratogenic and fetotoxic drugs and different exposure period  Additionally, Table 2 described maternal et fetal characteristics in case of exposure to teratogenic drugs. Whereas teratogenic drugs have been studied in preconceptional period and T1, maternal and fetal characteristics were reported for T2 and T3 in the previous submission. To be more relevant and understandable for the reader, we reported those characteristics in Supplementary Information (S3 Table). | *Not available* |
| Also note that some info is only supplied in Supplementary tables: mainly the names of medicine groups and whether they are fetotoxic or teratogenic. Therefore, the reader has no way of knowing immediately whats happening in Figure 1. | We paid attention to mention molecules and medicine groups in relation to the risk category in the legend of the Figure 1. | *Not available* |
| I quite like Figure 2 & 3 because it differentiates between teratogenic and fetotoxic meds. | We thank the reviewer for this comment. | *Not available* |
| A question- is it possible to provide a big picture graph for trends over the years for al harmful meds- wonder if that’s too many graphs- for fetotoxic and teratogenic together? You might choose to accept this comment or skip, but personally I feel it might give a neat overview of your findings. | It might be interesting. However, we choose to not provide a big graph with all medicine groups trends because there were to many lines, and it was not as readable as hoped. | *Not available* |
|  |  |  |
| **Discussion** |  |  |
| section for antiepileptics- useful to remind the reader of what this study found before comparing to international data. In line 428 the authors say the valproate use decreased dramatically- how much was it? | We thank the reviewer for this comment. Among antiepileptic drugs exposure, we found a marked decrease specifically for valproic acid (from 532 exposed pregnancies in 2013 to 83 exposed pregnancies in 2019 (from 6.7 to 1.2 per 10,000). Divalproate exposure decreased from 297 pregnancies in 2013 to 31 pregnancies in 2019 (from 3.8 to 0.4 per 10,000). We reminded the reader our results before comparing international data. | *Page 21, lignes 392-393* |
| The flow of discussion section needs work. It looks very hurried- perhaps the authors should spend more time finessing. An example: line 472: “Considering topical use, association with major congenital malformations among 215 exposed women has not been evidenced (47).” This sentence is incomplete and does not make sense. | We paid attention to rephrase this sentence. | *Page 23, lignes 438-441* |
| Also please remind the reader of your findings in the discussion every time the current study is referenced so the reader may identify the magnitude of difference between the existing literature and current study without having to go through all the tables. | We thank the reviewer for this point. We remind our results in the discussion every time we discuss about a drug prevalence. | *Page 22, ligne 427*  *Page 23, lignes 446-447* |
| Having said that the arguments used in the discussion section have improved greatly, from the previous version. However, the language needs some marked changes. | We did our best to rewrite the manuscript with better language and rephrase some paragraphs. | *Not available* |
|  |  |  |
| **Conclusion** |  |  |
| I feel the authors need to ‘sell’ this study better here- the study realy shows that the rates of systemic medicine use is comparable with the rest of the world, as opposed to what was initially imagined i.e. France has a higher use of harmful medicines. | We thank the reviewer for their advice and improve the conclusion. | *Not available* |
| The authorities need to keep a tab on NSAIDs- true, but the authors need to provide options of what can be done for women who are in genuine need of these meds? | We proposed that women need to better optimize their therapy before the starting of pregnancy, in relation with their physician. For chronic disease for e.g., the use of planning pregnancy consultation is an interesting initiative that could be enlarged. However, before proposing specific policies, our work did not allow to precisely characterize the use of NSAIDs in pregnancy (indication, type of women, etc…), which could be the purpose of further study. | *Not available* |
