# Supplementary material for: Trends in harmful drug exposure during pregnancy in France between 2013 and 2019: A nationwide cohort study
Source: PLoS One. 2024 Jan 10;19(1):e0295897. doi: 10.1371/journal.pone.0295897 (PMC10781191; doi:10.1371/journal.pone.0295897)
Supplement: Attachment — Submitted filename: Response to Reviewers.docx [file pone.0295897.s014.docx]

|  | **Response to Reviewers** | **Mention in the manuscript** |
| --- | --- | --- |
|  | | |
| **Overall Comments** | | |
| I feel the manuscript is in a much better condition than the previous versions. | We sincerely thank the reviewer for this second review and for her/his detailed and helpful comments. We are pleased to respond to each point. |  |
| Significant improvements have been made but it appears than your manuscript could benefit from editing by native english speaker. | As recommended, a native English speaker reviewed the manuscript. We sincerely hope that edits will improve the manuscript. |  |
| It still needs some editing for language.   - Keep tenses consistent. Suggest using past tense throughout. - One over 25 means little- better to use one ‘in’ 25. - Don’t capitalise drug names, stick to active voice throughout… etc. | We warmly thank the reviewer for helping us improving the manuscript.   - We corrected the text to use past tense throughout the entire manuscript. We only keep present tense in phrases which refer to general data such as those in - Introduction (first paragraph) - Methods (data source) - Discussion (in some paragraphs and phrases) - We corrected the manuscript sticking to active voice. - We corrected the expression “one over 25” to “one in 25”. - We corrected drug names if they were capitalized. |  |
|  |  |  |
| **Introduction:** |  |  |
| Can you give examples of manifestation of fetotoxity and teratogenicity in where you introduce the concepts? | We recognize that introduction of fetotoxicity and teratogenicity could benefit from examples. In the previous manuscript, we only mentioned the neurodevelopmental adverse effects of valproate and renal failure of NSAIDs. As recommended, we had an example of teratogenicity describing the thalidomide embryopathy characterized by phocomelia. | *Page 4, lines 61-62* |
| Do you know if the estimates of teratogenic medicine use in other countries included NSAIDs- perhaps say that somewhere? | Most previous studies used the FDA classification system to describe the teratogenic/harmful drug use. In this classification, NSAIDs used during the 3^rd^ trimester were classified in the category D, and they were included in the Swedish or Australian risk classifications systems. We completed introduction paragraph with this information. | *Page 4, lines 82 and 85* |
| Break down the paragraph on aims of study. Don’t say ‘related’ critical periods: it doesn’t mean much- I am thinking related to what? A suggestion is to write …”….we assessed exposure to… in the respective critical periods” or something to that effect. | We thank the reviewer for this issue. We followed your suggestion and improved the redaction for the paragraph of aims. | *Page 5, lines 91-94* |
|  |  |  |
| **Methods** |  |  |
| While the text describes what a CMUc is, it still doesmt say if only low income indviduals were included in this study?   - Yes, I understand that CMUc provides free access to healthcare for low income and SNDS is a national health insurance. But its not clear if the database used consisted of low income as well as others? - If yes, then did you study them separately? Would the data capture the drug use completely for all patients, or are there any differences in the data capture by population? - The authors say that it is used as a proxy for economic status- meaning that anyone without a CMUc affiliation (registration? Status?) will be a ‘general population’ category as opposed to low income? This aspect really targets generalisability of this study. | We thank the reviewer to encourage us to be more precise.   - The SNDS covers 98.9% of the French population and as such it includes patients with low income. We added this information. - In this paper, we described drug exposure for the overall population. We did not separate pregnancies according to mother’s characteristics. - Yes, the reviewer is right. In the overall population, we identified 4.6% of pregnancies with a CMUc affiliation which corresponds to a proxy of low-income status. The population without the CMUc affiliation corresponds to a population “without low-income status”. | *Page 6, lines 98 and 101-102* |
| Could you supply ICD 10 codes used in this study? As a supplementary? | We added ICD 10 and CCAM codes used to identify pregnancies outcomes in Supporting Information, S1 Table. Codes to identify maternal conditions were already reported in this table. | *Supporting Information, S1 Table* |
| Statistical analysis: please describe how you presented prevalence or exposure: as %? Although in some places it is n/10,000? Please harmonise this presentation. | We presented maternal characteristics, pregnancy outcomes and data related to the population in % (with 1 decimal point).  Regarding drug exposure, as some are very low, we presented it with prevalence as number per 10,000 pregnancies. We clarified this point in the method paragraph and harmonized data in the manuscript. | *Page 7, line 139* |
|  |  |  |
| **Results:** |  |  |
| Because you describe CMUc in the methods, good to describe the breakdown of the cohort by CMUc category in the first paragraph of results too. | We thank the reviewer for highlighting this issue. We added information on pregnancies with CMUc affiliation in the results and discussion paragraphs. | *Page 8, line 167*  *Page 20, lines 352-354* |
| 6.2% premature births- can you please say what gestational age you consider premature in parantheses- I can see the table says it clearly to be 37w. | We clarified this expression with adding definition for prematurity: “with 6.2% preterm births (before 37 weeks’ gestation)”. | *Page 9, line 169* |
| Please also specify the prevalence in this paragraph along with the overall numbers- because you describe it in the table. Its easy to see the exposed pregnancies belonged to women with much more comorbidities, more CMUc candidates too. | We thank the reviewer for this comment and specified if we described the overall population or the exposed pregnancies. | *Page 8, line 164* |
| Subheading teratogenic drugs: can you add absolute no. of pregnancies before the prevalence per 10,000 in parentheses for AEDs and topical retinoids- just to give context and to harmonise presentation of results. | We thank the reviewer for this comment. We added, when missing, number of exposed pregnancies, prevalence and harmonized the results and discussion. | *Page 15, lines 258 and 260* |
| Subheading fetotoxic drugs: the statement ….“We observed that exposure decreased from 94,740 pregnancies (181.8 per 10,000) during the second trimester, to 67,998 pregnancies (132.0 per 10,000) during the third trimester (Fig 1; S5 Table). Decrease mostly concerned NSAIDs for topical use and NSAIDs for systemic use”- while the absolute numbers decreased, the prevalence actually INCREASED from 181 to 132 per 10,000? You might want to revisit the calculations. I fear NSAID prevalence might have increased too? This might change your conclusions. Best to use the prevalence rates to describe decrease or increase and absolute numbers in parantheses to avoid confusion. | We recognize that the sentence wasn’t as clear as needed. We rephrased with “We observed a decrease from 94,740 (182 per 10,000) exposed pregnancies during the second trimester, to 67,998 (132 per 10,000) exposed pregnancies during the third trimester (Fig 1; S5 Table).”.  Prevalence of 182 per 10,000 pregnancies correspond to the 94,740 exposed pregnancies during the second trimester in relation to the 5,210,419 pregnancies that have been identified during the second trimester.  Prevalence of 132 correspond to the 67,998 exposed pregnancies during the third trimester in relation to the 5,149,745 pregnancies that have been identified during the third trimester. | Page 13, lines 209-211 |
| It is clear the use of antiepileptics and statins were also very high. Can you comment on which ones in the paragraph on teratogenic meds? The information could be supplied here readily from the supplementary files and will add depth to your arguments. | As recommended, we added information about retinoids, teratogenic antiepileptics and statins. | *Page 16, lines 260-261, and 265-267.* |
| Subheading on trends: In the statement “Regarding exposure to retinoids for systemic use, it decreased from 107 pregnancies (1.4 per 10,000) in 2013 to 81 exposed pregnancies during preconceptional period or T1”- do you mean to add a calendar year here? Also please add prevalence next to 81 pregnancies/\. | The number of 81 referred to the number of pregnancies exposed in 2019. We rephrased the sentence and added prevalence. | *Page 15, line 260* |
| Can I ask the reason to separate valproic acid and divalproate? Its not common to see them differentiated. If they have similar effect on congenital defects then maybe keep them together and report the prevalence as one drug. | We acknowledge that the two drugs are very similar. However, they had different indications and the database allowed to individualize them. We though that this information will be useful to the reader. |  |
| Figure 2, subfigure “(B) Any teratogenic drug, excluding retinoids for topical use”- It is repeatition of fgure (A) just without topical retinoids. My suggestion in the first review was to provide a separate line trend for OVERALL teratogenic use with and without topical retinoids. I think a trendline is required for overall use here and in the next figure for fetotoxic drugs | We thank the reviewer to rephrase his/her comment and apologize for the misunderstood. In figure 2, we provided a separated graph with overall teratogenic exposure with and without topical retinoids. In figure 3, separated graph with overall fetotoxic exposure with and without topical NSAIDs. | *Figure 2*  *Figure 3* |
| Thanks for adding the sensitivity analysis- this is great! But please stick to the same presentation for prevalence: Percentage or n/10,000. | We chose “number per 10,000 pregnancies” to present drug exposure prevalence in the entire manuscript. |  |
| % with 2 decimal points is too much to read! | We changed data into 1 decimal point to enhance readability of the manuscript and Supporting Informations. | *Manuscript Pages 10, 11 and 12*  *Supporting Information, S3, and S8-10* |
|  |  |  |
| **Discussion** |  |  |
| “Among maternal characteristics, women with comorbidities such as psychiatric disorders,354 diabetes or hypertension, were more likely to be exposed to a harmful drug during pregnancy.” I don’t think you have provided risk ratios here. Please avoid the use of term likely. Change to say cohort consisted of higher proportion of xyz compared to the pqrst cohort etc. | We thank the reviewer for this relevant point and rephrased the sentence as recommended. | *Page 20, line 350* |
| In our study, prescriptions for valproic derivatives dramatically decreased from 2013 to 2019, especially in the 2013-2017 period”- revisit phrasing of this sentence | We paid attention to rephrase this sentence. | Page 22, lines 397-399 |
| Since the valproate derivatives harmful effects mediatisation”- I am not sure if mediatasation is a word- what do you want to say- was it in the media a lot? Were there policy changes then? Please describe clearly | We also paid attention to modify this sentence. | Page 22, lines 389-390 |
| In the paragraph on AEDs   - while you describe all the literature, please make a comment on how your estimates relate to these international estimates- higher lower same or cant comment? - Also no need to comment on age in the last sentence because tour study doesn’t consider it. - How did the measures work in your case- your argument is that the measures came into effect in 2014-15 and there was decrease in use in Switzerland 2015-18. - Your study was 2013 until 2019- what do you have to say about the impact off the EMA measures on use in France? This paragraph needs revisiting. | - We thank the reviewer for this comment. We added that our manuscript confirms the decrease trend in valproic acid prescriptions. - As recommended, we did not comment on age. - Without statistic tests, we described that valproic derivatives prescriptions decreased after the worldwide recognition of the valproate harmful effects and the EMA recommendations. We rephrased the related sentences. - Our findings suggested that the EMA measures had a beneficial impact in France | *Page 22, lines 398-399*  *Page 22, lines 403-404* |
| - NSAIDs: “Exposure is lower in Northern Europe such as in Sweden and Norway where it ranges from 0.3% in second trimester to 0.1% in third trimester (31,40). NSAIDs exposure is thus higher in France.”- nowhere before this sentence have you described the numbers in France. Perhaps where you say what your study found say what % or prevalence you found? | We thank the reviewer for this comment and rephrased this paragraph. NSAIDs exposure was higher in France than in Northern countries. But our study described a lower exposure than another French study based on a national sample which reporting data on a previous period. Lower exposure in our study was thus in accordance to decrease trend over the years. | *Page 23, lines 418-427* |
| Strengths and limitations: • Perhaps add a limitation that some pregnancy durations were estimated using gestational age assessment at birth- this could have caused some over- or under-estimation of exposure based on estimation of GA? | We sincerely thank the reviewer for this discussion. There were two methods to estimate the date of conception and duration of pregnancy. This issue might induce an ambiguity. However, our results regarding gestational age at birth are strictly concordant with national epidemiological data. |  |
|  |  |  |
| **Conclusion** |  |  |
| Please make a comment regarding change in teratogenic and fetotoxic medicine use over years here- its great information! | We thank the reviewer for their advice and improve the conclusion. | *Page 26, lines 481-482* |
