# Supplementary material for: Trends in harmful drug exposure during pregnancy in France between 2013 and 2019: A nationwide cohort study
Source: PLoS One. 2024 Jan 10;19(1):e0295897. doi: 10.1371/journal.pone.0295897 (PMC10781191; doi:10.1371/journal.pone.0295897)
Supplement: Attachment — Submitted filename: Response to Reviewers.docx [file pone.0295897.s015.docx]

|  | **Response to Reviewers** | **Mention in the manuscript** |
| --- | --- | --- |
|  | | |
| **Overall Comments** | | |
| **Please correct a few grammatical errors and sentences difficult to read** | We sincerely thank the reviewer for his/her response, and we are pleased to correct the manuscript. |  |
|  |  |  |
| **Introduction:** |  |  |
| Lignes 68–70, Teratogenic drug exposure stopped shortly before conception is also of concern given the possible persistence of the drug in the body during early embryogenesis, perhaps before the mother becomes aware she is pregnant. |  | *Page 4, lines 68-70* |
|  |  |  |
| **Discussion** |  |  |
| - Ligne 449, prescription was, not prescriptions was; - Ligne 458, or, not nor; - Lignes 463–464, Similarly, NSAID prescriptions may have been filled during the third trimester in anticipation of use to relieve postpartum pain; - Ligne 484: underline |  | *Page 24, line 449*  *Page 24, line 458*  *Page 25, lines 463-464*  *Page 25, line 484* |
|  |  |  |
